# Optical Control of GABA_A_ Receptors with a Fulgimide‐Based Potentiator†

**DOI:** 10.1002/chem.202000710

**Published:** 2020-09-11

**Authors:** Karin Rustler, Galyna Maleeva, Alexandre M. J. Gomila, Pau Gorostiza, Piotr Bregestovski, Burkhard König

**Affiliations:** ^1^ Institute of Organic Chemistry Department of Chemistry and Pharmacy University of Regensburg 93053 Regensburg Germany; ^2^ INSERM, INS Institut de Neurosciences des Systèmes Aix-Marseille University 13005 Marseille France; ^3^ Institute for Bioengineering of Catalonia (IBEC) The Barcelona Institute of Science and Technology Barcelona 08028 Spain; ^4^ Institució Catalana de Recerca i Estudis Avançats (ICREA) 08020 Barcelona Spain; ^5^ Network Biomedical Research Center in Biomaterials Bioengineering and Nanomedicine (CIBER-bbn); ^6^ M. Sechenov First Moscow State Medical University Moscow Russia; ^7^ Institute of Neurosciences Kazan State Medical University Kazan Russia

**Keywords:** fulgimides, GABAA Receptors, in vivo, photopharmacology

## Abstract

Optogenetic and photopharmacological tools to manipulate neuronal inhibition have limited efficacy and reversibility. We report the design, synthesis, and biological evaluation of Fulgazepam, a fulgimide derivative of benzodiazepine that behaves as a pure potentiator of ionotropic γ‐aminobutyric acid receptors (GABA_A_Rs) and displays full and reversible photoswitching in vitro and in vivo. The compound enables high‐resolution studies of GABAergic neurotransmission, and phototherapies based on localized, acute, and reversible neuroinhibition.

## Graphical abstract

**Fulgazepam**: Herein, the design, synthesis, and biological evaluation of Fulgazepam, a fulgimide derivative of benzodiazepine that behaves as a pure potentiator of ionotropic γ‐aminobutyric acid receptors (GABA_A_Rs) and displays full and reversible photoswitching in vitro and in vivo, is reported.

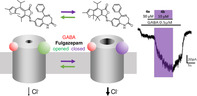

In the quest to understand brain circuits, controlling neuronal activity with light has become an essential tool to manipulate the balance between excitation and inhibition.^1^ However, optogenetic tools to inhibit neurons (halorhodopsin pumps, anion‐conducting bacterial channelrhodopsins and chloride‐conducting ChR2 mutants)^2^, ^3^, ^4^ have very limited conductance and dynamic responses to depolarization. Caged γ‐aminobutyric acid(GABA) compounds have been used to inhibit spines and to control seizures, but uncaging is irreversible and their neurotoxicity^5^ has been avoided only recently by means of allosteric ligands.^6^ A powerful alternative is using reversible chemical photoswitches^7^, ^8^, ^9^, ^10^ to harness endogenous anion‐conducting receptor‐channels like GABA and glycine receptors (GABA_A_R, GlyR), which mediate inhibitory neurotransmission in the mammalian central nervous system.^11^ Although some GABA_A_R photoswitches have been reported based on azobenzene,^12^, ^13^, ^14^ this photochromic group displays several shortcomings: it provides incomplete photoconversion due to a substantial overlap of the absorption maxima of *cis* and *trans* isomers, and can alter the pharmacophore activity. Indeed, in all azobenzene derivatives of benzodiazepines (allosteric potentiators of GABA_A_R) this characteristic property is abolished, as found in the 7‐amino site of nitrazepam, which is reportedly tolerant of other substitutions.^15^ In addition, GABA_A_R photoswitches described so far are agonists or antagonists that interfere with endogenous neurotransmission, not pure modulators.^13^, ^16^, ^17^

Here we introduce Fulgazepam (compound **4**), a derivative of diazepam based on a different photochromic group (fulgimide), which shows both quantitative reversible switching and GABA_A_R potentiation. It is inactive in its open isomer and potentiates the receptor in its closed isomer without concomitant agonist or antagonist activity, thus overcoming the above mentioned hurdles and displaying an ideal photopharmacological profile. Most importantly, Fulgazepam works both in vitro and in vivo, which indicates that the compound is devoid of toxicity and has favourable molecular properties enabling wide applications.

In contrast to azobenzenes, dithienylethenes, fulgides and their fulgimide‐named amide derivatives generally feature high photostationary states (PSS) with both photoisomers being thermally stable.^7^, ^18^ As dithienylethenes often lack of switching efficiency and stability in polar solvents due to a twisted intramolecular electron charge transfer,^20^, ^21^, ^22^ we chose fulgi(mi)des as photochromic scaffold in this study. Both subtypes can be interconverted between their flexible, less‐coloured ring‐open and their rigid, more coloured ring‐closed isomer upon light‐induced conrotatory 6π‐electrocyclic rearrangement (Figure 1, Scheme 1).^18^, ^23^ Although switching from the open to the closed form is usually triggered using UV light, this might be avoided by the isolation and separate application of both isomers. In addition, this ensures the application of quantitative amounts of either the open or the closed form. Thereby, a biological effect can clearly be assigned to one or the other conformation. Synthetic investigations revealed the beneficial effects of an isopropyl group in the alpha bridge position of the fulgide, as the *E–Z* isomerization of the open isomer is suppressed due to steric hindrance and consequently only two distinct isomers are observed (Figure 1 A).^19^

**Figure 1:**
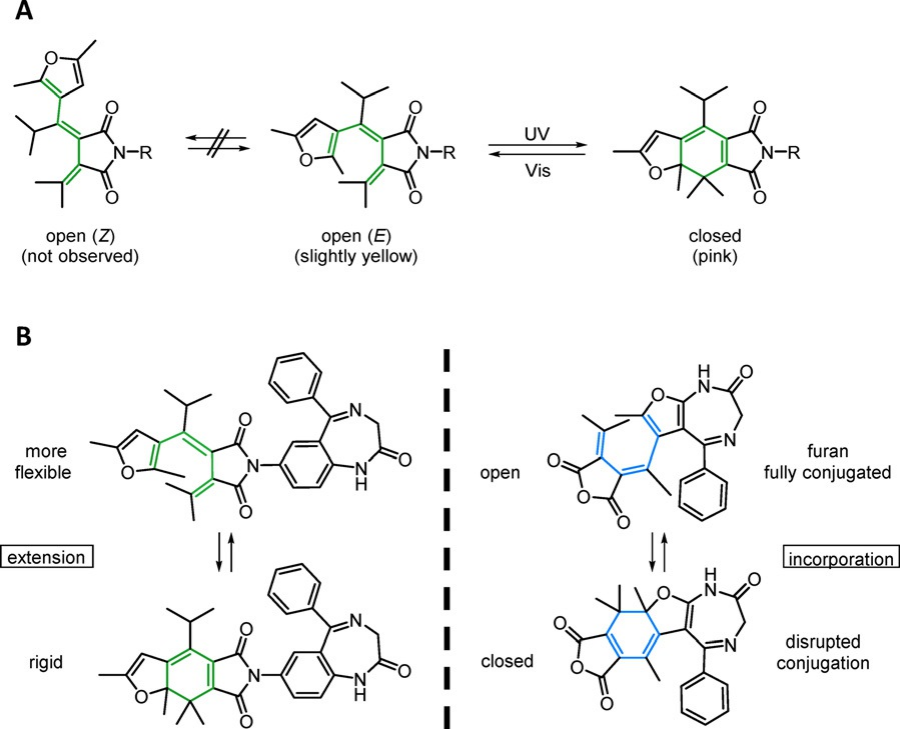
A) Furan‐fulgimide in its open and closed isomeric state interconvertible by illumination with UV and visible light.^18^, ^19^ B) Left: Pharmacophore nitrazepam and its extension towards a photochromic fulgimide. Right: Derivatization towards a photochromic diazepine fulgide hybrid.

One advantage of fulgimides over fulgides is their improved switching in aqueous solutions and high stability. Furthermore, the two‐step transformation of fulgides towards fulgimides via nucleophilic ring‐opening of the anhydride by a primary amine and subsequent recyclization allows the smooth introduction of amino‐functionalized biomolecules.^18^, ^19^ However, few biological applications of fulgi(mi)des are reported.^24^, ^25^, ^26^ The transformation of a known ligand into a photoresponsive molecule can be designed by either extending the pharmacophore with a photoswitch or via incorporation of the photochromic scaffold as part of the drug's chemical structure. Once introduced, ideally one isomeric state is biologically active whereas the other loses its required interactions. In the presented work, both approaches were pursued. On the one hand, a furan‐fulgide photochromic scaffold was merged with an amino‐benzodiazepine under fulgimide formation (Figure 1 B, left panel). A difference in activity can be expected from the different flexibility of the isomeric states. On the other hand, a functionalized diazepine was synthesized aiming for a photochromic benzodiazepine core (Figure 1 B, right panel). In this case, the difference in activity was expected to be given by the different conjugation of the pharmacophore's aromatic system upon switching. Unfortunately, the latter modified pharmacophore (compound **9**, Figure 2 B) was inactive in patch‐clamp studies (data not shown) and the synthesis towards the photoswitch was not further pursued.

**Figure 2:**
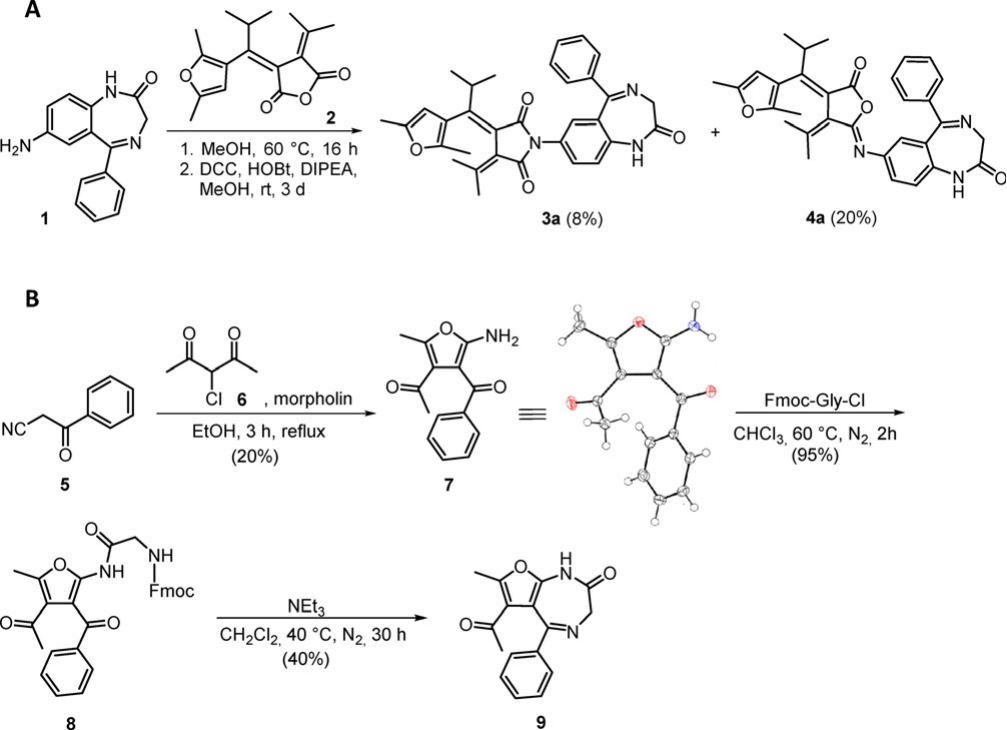
A) Synthesis of fulgimide‐nitrazepam **3 a** and its iso‐fulgimide derivative **4 a**. We performed the reaction of furano‐fulgide **2** 25 with amino‐nitrazepam **1** 27 upon addition of dicyclohexylcarbodiimide (DCC), diisopropylethylamine (DIPEA), and 1‐hydroxybenzotirazole (HOBt) in methanol, to afford the desired benzodiazepine‐furano‐fulgimide **3 a** and its iso‐fulgimide derivative **4 a**. B) Synthesis of the highly functionalized furan **7** and its diazepine formation towards compound **9**.^21^, ^24^, ^25^, ^26^

To obtain a photochromic pharmacophore core, we envisioned a functionalized diazepine derivative (**7**) providing an acetyl group in position 3 required for Stobbe^28^, ^29^, ^30^ condensation towards fulgide formation and a methyl‐group in position 2 beneficial for the fulgide's switching performance.^25^ For diazepine formation, the highly functionalized precursor **7** requires in addition a primary amine in position 5 and a phenone substitution in position 4.^27^ Based on the literature known Gewald‐reaction^31^ and screening of solvents and bases the desired functionalized furan **7** was obtained in good yield in a one‐step synthesis starting from commercially available benzoylacetonitrile **5** and 3‐chloroacetylaceton **6** (Figure 2 B).^32^, ^33^ The following ring closure required for diazepine formation of **9** was performed in analogy to literature reports.^27^

Regarding the photochromic properties, the introduction of the bulky isopropyl group on the 1,3,5‐hexatriene system of the fulgide avoided the undesired UV light induced *E–Z* isomerization of the open *E*‐fulgimide isomer (Figure 1 A). Only the *E* isomer undergoes a photocyclization reaction to the thermally stable closed isomer (Figure 3 A). The colorless open isomers **3 a** and **4 a** were converted to their strongly colored ring closed isomers **3 b** and **4 b** upon illumination with UV light of *λ*=365 nm. The absorption maximum of the open isomer around 340 nm decreased and a new maximum around 520 nm representing the closed isomer formed (Figure 3 B). Both compounds show almost quantitative ring‐closing (93 % for **3 b** and 95 % for **4 b**, measured 50 μm in DMSO) and quantitative ring‐reopening using green light (*λ*=505 nm or 528 nm).

**Figure 3:**
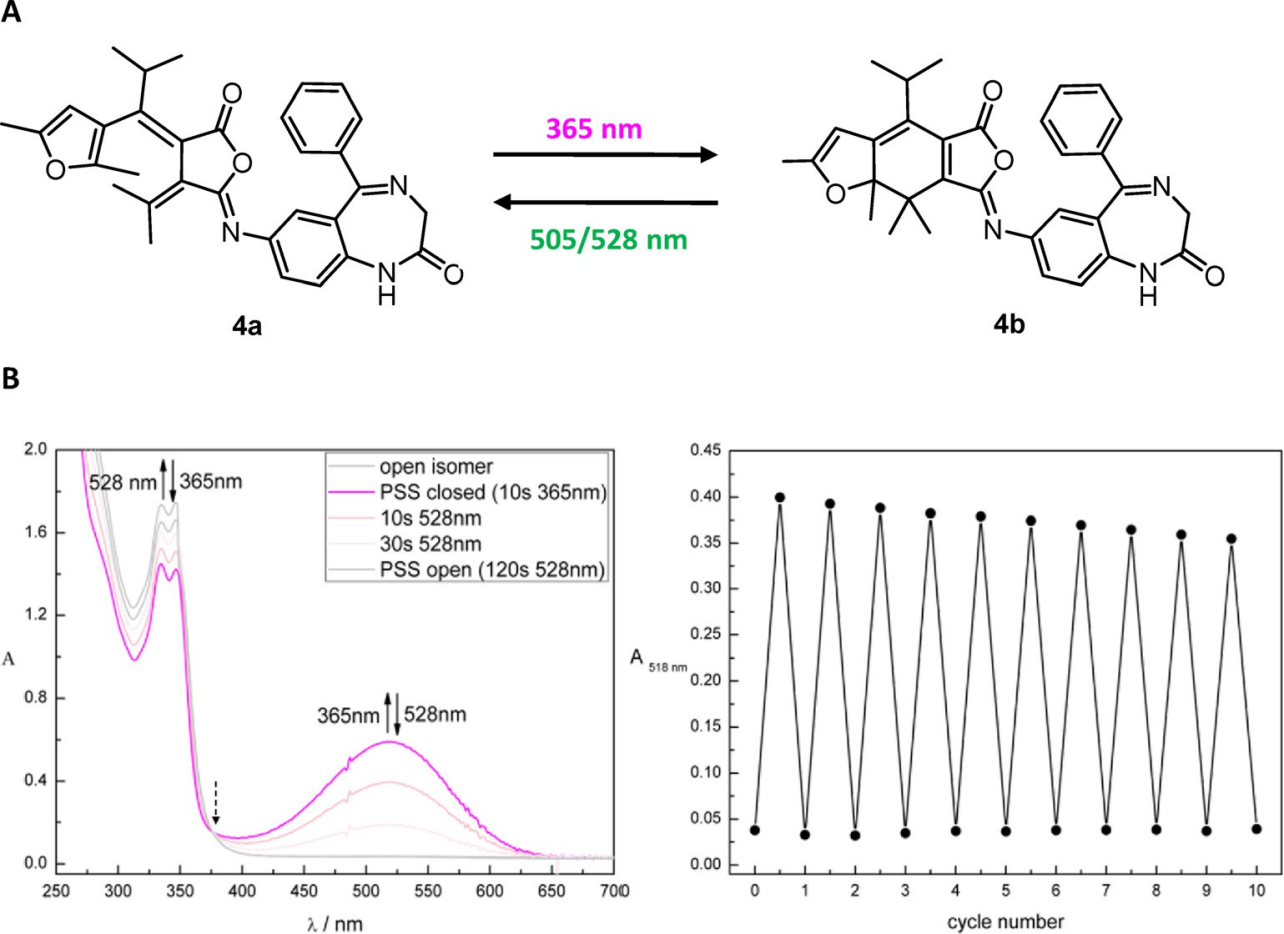
A) Illumination induced ring‐closing (**4 b**) and ring‐opening (**4 a**) of iso‐fulgimide **4** (Fulgazepam). B) Photochromic properties of iso‐fulgimide **4** (50 μm) measured in DMSO. Left: Spectral evolution of **4 a** (open isomer; grey spectrum) upon illumination with 365 nm and re‐opening of **4 b** (closed isomer; purple spectrum) upon illumination with 528 nm. Right: Cycle performance of **4** upon alternate illumination with 365 nm (ring closing) and 528 nm (ring opening) detected at 518 nm (*λ*_max_ closed isomer). UV–Vis absorption spectrum and cycle performance of isofulgimide **4** upon illumination with 365 nm and 505 nm. Black arrows indicate the spectral evolution upon illumination. Dotted black arrows label isosbestic points indicating a clear two component switching. After 10 s illumination at *λ*=365 nm the closed‐PSS was reached and 93 % of the closed‐isomer accumulated. Quantitative reopening was achieved within 120 s illumination at *λ*=505 nm or 528 nm, respectively. Both compounds show sufficient fatigue resistance for photopharmacological experiments over ten measured cycles upon alternate illumination with 365 nm for closing and 528 nm for opening.

To characterize Fulgazepam in vitro, patch clamp experiments were performed on cells transiently expressing α_1_β_2_γ_2_ subunits of the GABA_A_ receptor. This receptor possesses the canonical benzodiazepine allosteric site and its EC_50_ for GABA is about 8 μm.^14^ The effects of the fulgimide‐based benzodiazepine derivatives **3** and **4** on the receptor's function were studied upon co‐application of 0.5 μm GABA, that is, a concentration below the EC_50_ (close to EC_3_) that allows to observe allosteric potentiation of GABA_A_R‐mediated currents.^34^

Application of compound **4 a** (open isomer) (10 μm) caused no significant effect on GABA_A_‐mediated currents, while application of **4 b** (closed isomer), generated by pre‐illumination with UV light (365 nm), induced an increase of GABA_A_‐mediated current amplitudes (Figure 4 A). Thus, the isomers of compound **4** interact differently with GABA_A_Rs, being inactive in the open form and potentiating in the closed form. Analysis of a series of dose‐response curves established that the EC_50_ for **4 b** was 13 μm (*n=*6; Figure 4 B). Figure 4 C demonstrates that UV illumination switches the conformation of 10 μm compound **4** (from **4 a** to **4 b**) and increases the amplitude of GABA‐induced currents by 228±41 % (Figure 4 D; *n=*11). Compound **3 a** in its open state co‐applied with GABA (0.5 μm) induced a strong potentiation of GABA_A_R‐mediated currents (Figure S3A). This potentiation was not sensitive to illumination by UV light and subsequent isomerization to the closed isomer **3 b** (Figure S3 B) and the kinetics of compound **3 b's** development (slow wash‐in and slow wash‐out) was similar to the one of **4 b**. Application of 10 μm of **3 a** increased the current amplitude by 292±65 %, while 50 μm of **3 a** increased the current amplitude by 544±107 % (*n=*11). The EC_50_ of **3 a** was 12 μm, similar to the one of compound **4 b** (Figure S3 C, *n=*11). The degree of GABA_A_R potentiation induced by **3 a** markedly varied for different cells (*cf*. A and B in Figure S3). A similar behavior was observed for **4 b**. We suggest that this effect reflects the variability in the EC_50_ of GABA in different cells, as it has been shown that allosteric potentiation decreases at high GABA concentrations.^35^

**Figure 4:**
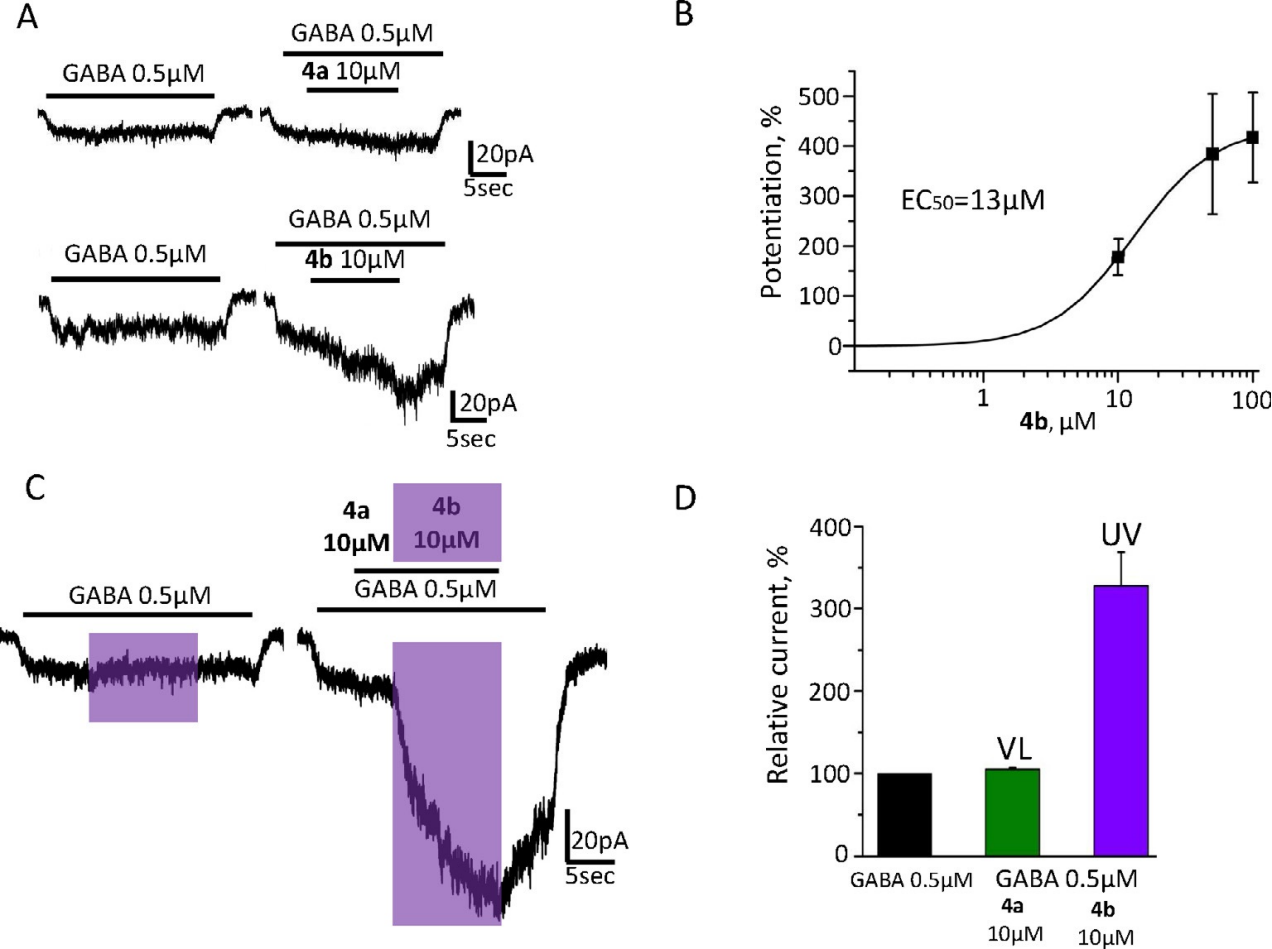
The effect of compounds **4 a** and **4 b** on GABA_A_‐mediated currents. A) Upper panel: representative traces of currents induced by application of GABA 0.5 μM and by mixture of GABA 0.5 μM with **4 a** 10 μM; lower panel: representative traces of currents induced by application of GABA 0.5 μM and by mixture of GABA 0.5 μM with **4 b** 10 μM. Durations of applications of GABA and compound **4** are indicated by black bars above the traces. B) Cumulative dose‐response curve for compound **4 b** (*n=*6). C) Representative traces demonstrating the effect of **4 a** photoswitching on the amplitude of GABA‐induced currents. On the left: current was induced by application of GABA 0.5 μM; on the right: at the same trace current was induced subsequently by GABA 0.5 μM, by mixture of GABA with **4 a** 10 μM under visible light and upon illumination with UV light (**4 b**). Duration of UV illumination is indicated by a violet rectangle. Note the prominent increase of the GABA‐induced current in the presence of **4** during illumination with UV light, which triggers ring‐closing (**4 b**). D) Cumulative graph representing mean relative amplitude of currents induced by application of GABA 0.5 μM (black column), GABA 0.5 μM+**4 a** 10 μM (green column) and GABA 0.5 μM+**4 b** 10 μM (violet column) upon illumination with UV light (*n=*11).

The outstanding photopharmacological profile of Fulgazepam (**4**) as GABA_A_R potentiator prompted further assays in vivo. Studies in zebrafish larvae show that the compound alters their behavior depending on isomerization and that this effect can be maintained over time in the absence of illumination. As both compound states **4 a** and **4 b** are stable in the dark, larvae behaviors could be studied using pre‐illuminated compounds in the dark followed by in situ illumination using 365 and 500 nm wavelengths (Figure 3). Pre‐illuminated compound **4 b** altered in a dose dependent manner the behavior of undisturbed larvae. In particular,100 μm **4 b** evoked an increase in swimming distance (Figure 5 B, top). The effect of subsequent illumination was also studied. For all concentrations of **4 a**, UV light (hence, photoconversion to **4 b**) significantly increased larvae motility, and this effect was potentiated during the following dark period and reduced to vehicle levels upon illumination with visible light (Figure 5 A and Figure 5 B, bottom). Therefore, these changes in larvae motility are triggered by conformational changes of compound **4** rather than by natural photoresponsive behaviors. A significant increase in larvae activity, above vehicle levels, is observed when **4 b** isomerisation is potentiated (during and after UV illumination) and lowers to natural activity when **4 a** is recovered with green illumination independently of the initial Fulgazepam state that is administered to larvae.

**Figure 5:**
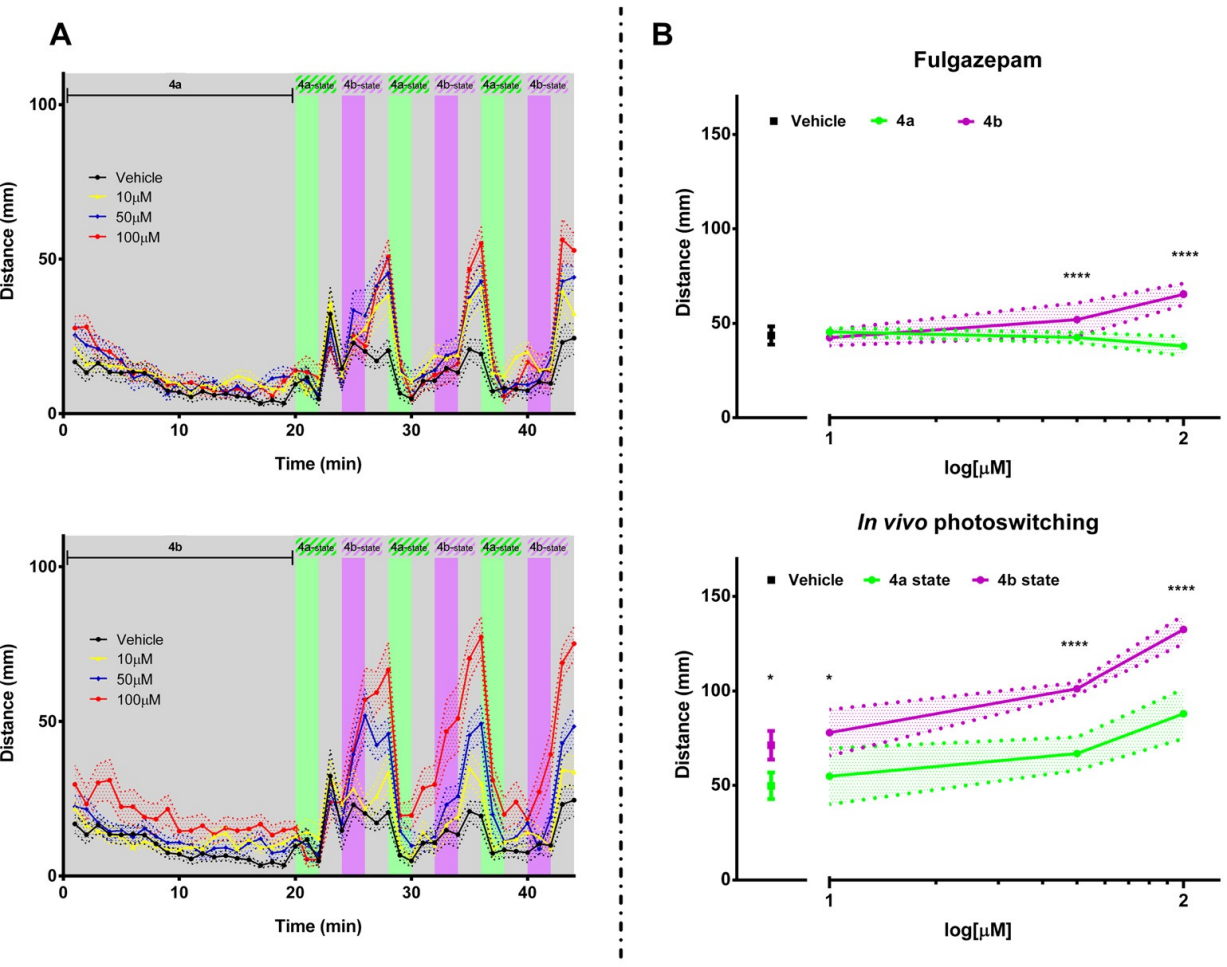
A) Effect of Fulgazepam in wild type zebrafish larvae. One‐minute trajectories of average swimming distances (*n=*12 per treatment) are shown for vehicle (1 % DMSO) and three different concentrations of compound **4**, starting with pre‐irradiated solutions of **4 a** (top) and **4 b** (bottom). For the first 20 minutes, larvae were undisturbed in complete darkness (relaxation period, RP), therefore maintaining compound **4** stable states. Following RP larvae were illuminated with three consecutive cycles of visible light (500 nm) and UV (365 nm) with discrete dark between each wavelength. Colored areas show standard error of the mean (S.E.M.). B) Top: Quantification of swimming distances over the last 5 minutes of the RP (darkness) from two independent experiments (*n=*24 per treatment) for both pre‐illuminated compounds **4 a** (green trace) and **4 b** (violet trace) and vehicle (1 % DMSO). Bottom: Quantification of total distance swam after light periods (UV and visible light) for compound **4** and vehicle (*n*=12 per treatment). *p‐value<0.05, ****p‐value<0.0001. Colored areas show standard deviation (S.D.).

In summary, we have achieved the functionalization of the benzodiazepine nitrazepam via extension by a fulgimide and report a new photochromic potentiator of GABA_A_Rs. The synthesized fulgimides **3** and **4** (Fulgazepam) display good photochromic properties and high photostationary states. Both fulgimides preserve the GABA_A_R potentiator behavior that is characteristic of benzodiazepines, indicating that it is a pharmacologically tolerable substitution, in contrast to azobenzenes at the same position. Remarkably, both fulgimides are photochromic but only Fulgazepam (**4**) enables controlling the pharmacological activity with light. The open conformation of Fulgazepam (**4 a**) does not influence the amplitude of GABA_A_R currents in vitro, while switching to its closed form **4 b** with UV light strongly potentiates them. The open (**4 a**) and closed (**4 b**) conformation of iso‐fulgimide **4** produce different behavioral outcomes in zebrafish larvae. The ring‐open isomer **4 a** does not alter larvae swimming activities, neither applied directly nor obtained by illumination cycles, and the closed conformation **4 b** increases larvae motility in a dose dependent manner both during prolonged dark periods and under UV illumination. Hence, Fulgazepam photoswitching reversibly controls the behavior of larvae, producing high activity swimming upon UV illumination, which persists for continuous dark periods, and reducing activity to control levels with visible light illumination.

GABA_A_Rs mediate fast inhibition of neural activity and are determinant in cognition, learning, and memory.^36^, ^37^, ^38^ Malfunction of these receptors leads to epilepsy, anxiety, depression and sleep disorders.^37^ Clinical treatments with GABA_A_R modulators have limited efficacy and adverse side effects,^39^, ^40^ which could be alleviated by targeting drug action at specific circuits or locations.

We have recently developed an azobenzene‐nitrazepam based compound (Azo‐NZ1) that allows photo‐modulation of GABA_A_ receptors.^14^ In *trans*‐configuration, this compound selectively interacts with the chloride‐permeable channel causing inhibition of GABA‐induced currents, while UV‐induced transition to the *cis*‐state results in channel unblocking and restoring the current amplitude. Such unexpected action for a derivative of benzodiazepine (a GABA_A_R potentiator) was explained by molecular docking calculations and mutagenesis analysis indicating that the 2′ residue of the channel‐forming transmembrane TM2 domain is the target of Azo‐NZ1 blocking action.

In contrast to Azo‐NZ1, the GABA_A_R modulator presented here (Fulgazepam) does produce potentiation of GABA‐induced currents. This GABA_A_R photoswitch displays unique characteristics as a direct result of its photochromic (fulgimide) and pharmacological (diazepam) moieties: (i) the fulgimide scaffold imparts complete reversible switching of Fulgazepam's conformation; (ii) both Fulgazepam states are stable and can be readily obtained by illumination with light of the appropriate wavelengths; (iii) Fulgazepam is sufficiently soluble in aqueous solution and effectively photocontrols endogenous GABA_A_Rs in vitro–in its closed form it is a pure potentiator of GABA_A_Rs without agonist or antagonist activity; (iv) Fulgazepam does not display toxicity in zebrafish and allows controlling their behavior with light. These outstanding molecular properties enable dissecting the mechanisms of GABAergic neurotransmission at high spatiotemporal resolution, and pursuing novel phototherapies based on localized, acute, and reversible neuroinhibition.

## Experimental Section

All animal experiments were conducted according to the EU Directive 2010/63/EU on the protection of animals used for scientific purposes. According to this directive, zebrafish are considered vertebrates and therefore subject to legislation governing animal testing, but embryos and non‐independently feeding form larvae are excepted.

## Conflict of interest

The authors declare no conflict of interest.

## Supporting information

As a service to our authors and readers, this journal provides supporting information supplied by the authors. Such materials are peer reviewed and may be re‐organized for online delivery, but are not copy‐edited or typeset. Technical support issues arising from supporting information (other than missing files) should be addressed to the authors.

Supplementary

## References

[1] O. Yizhar , L. Fenno , M. Prigge , F. Schneider-Warme , T. Davidson , D. O′Shea , V. Sohal , I. Goshen , J. Finkelstein , J. Paz , K. Stehfest , R. Fudim , C. Ramakrishnan , J. Huguenard , P. Hegemann , K. Deisseroth , Nature 2011, 477, 171–178. 21796121 10.1038/nature10360 PMC4155501

[2] E. G. Govorunova , O. A. Sineshchekov , H. Li , J. L. Spudich , Annu. Rev. Biochem. 2017, 86, 845–872. 28301742 10.1146/annurev-biochem-101910-144233 PMC5747503

[3] E. G. Govorunova , O. A. Sineshchekov , R. Janz , X. Liu , J. L. Spudich , Science 2015, 349, 647–650. 26113638 10.1126/science.aaa7484 PMC4764398

[4] J. Wietek , J. S. Wiegert , N. Adeishvili , F. Schneider-Warme , H. Watanabe , S. Tsunoda , A. Vogt , M. Elstner , T. Oertner , P. Hegemann , Science 2014, 344, 409–412. 24674867 10.1126/science.1249375

[5] X. Yang , D. L. Rode , D. S. Peterka , R. Yuste , S. M. Rothman , Ann. Neurol. 2012, 71, 68–75. 22275253 10.1002/ana.22596 PMC4133113

[6] L. Sansalone , J. Bratsch-Prince , S. Tang , B. Captain , D. D. Mott , F. M. Raymo , Proc. Natl. Acad. Sci. USA 2019, 116, 21176–21184. 31575739 10.1073/pnas.1902383116 PMC6800354

[7] W. Szymański , J. M. Beierle , H. A. V. Kistemaker , W. A. Velema , B. L. Feringa , Chem. Rev. 2013, 113, 6114–6178. 23614556 10.1021/cr300179f

[8] W. A. Velema , W. Szymanski , B. L. Feringa , J. Am. Chem. Soc. 2014, 136, 2178–2191. 24456115 10.1021/ja413063e

[9] K. Hüll , J. Morstein , D. Trauner , Chem. Rev. 2018, 118, 10710–10747. 29985590 10.1021/acs.chemrev.8b00037

[10] J. Broichhagen , J. A. Frank , D. A. Trauner , Acc. Chem. Res. 2015, 48, 1947–1960. 26103428 10.1021/acs.accounts.5b00129

[11] T. Lynagh , S. A. Pless , Front. Physiol. 2014, 5, 160. 24795655 10.3389/fphys.2014.00160 PMC4006026

[12] R. Huckvale , M. Mortensen , D. Pryde , T. G. Smart , J. R. Baker , Org. Biomol. Chem. 2016, 14, 6676–6678. 27327397 10.1039/c6ob01101b

[13] L. Yue , M. Pawlowski , S. S. Dellal , A. Xie , F. Feng , T. S. Otis , K. S. Bruzik , H. Qian , D. R. Pepperberg , Nat. Commun. 2012, 3, 1095. 23033071 10.1038/ncomms2094 PMC4023869

[14] G. Maleeva , D. Wutz , K. Rustler , A. Nin-Hill , C. Rovira , E. Petukhova , A. Bautista-Barrufet , A. Gomila-Juaneda , P. Scholze , F. Peiretti , M. Alfonso-Prieto , B. König , P. Gorostiza , P. Bregestovski , Br. J. Pharmacol. 2019, 176, 2661–2677. 30981211 10.1111/bph.14689 PMC6609548

[15] A. M. J. Gomila , K. Rustler , G. Maleeva , A. Nin-Hill , D. Wutz , A. Bautista-Barrufet , X. Rovira , M. Bosch , E. Mukhametova , M. Mukhamedyarov , F. Peiretti , M. Alfonso-Prieto , C. Rovira , B. König , P. Bregestovski , P. Gorostiza , bioRxiv 2019, 744391.

[16] M. Stein , S. J. Middendorp , V. Carta , E. Pejo , D. E. Raines , S. A. Forman , E. Sigel , D. Trauner , Angew. Chem. Int. Ed. 2012, 51, 10500–10504; 10.1002/anie.201205475 PMC3606271 22968919

[17] M. T. Richers , J. M. Amatrudo , J. P. Olson , G. C. R. Ellis-Davies , Angew. Chem. Int. Ed. 2017, 56, 193–197; 10.1002/anie.201609269 PMC5195861 27910251

[18] C. Brieke , F. Rohrbach , A. Gottschalk , G. Mayer , A. Heckel , Angew. Chem. Int. Ed. 2012, 51, 8446–8476; 10.1002/anie.201202134 22829531

[19] F. Strübe, *Synthese photochromer Fulgide: photoschaltbare Fluoreszenz und ultraschnelle Reaktionen*, Bielefeld University, **2011**.

[20] T. Yamaguchi , K. Uchida , M. Irie , J. Am. Chem. Soc. 1997, 119, 6066–6071.

[21] M. Irie , K. Sayo , J. Phys. Chem. 1992, 96, 7671–7674.

[22] C. Fleming , P. Remón , S. Li , N. A. Simeth , B. König , M. Grøtli , J. Andréasson , Dyes Pigm. 2017, 137, 410–420.

[23] A. Santiago , R. S. Becker , J. Am. Chem. Soc. 1968, 90, 3654–3658.

[24] N. A. Simeth , L.-M. Altmann , N. Wössner , E. Bauer , M. Jung , B. König , J. Org. Chem. 2018, 83, 7919–7927. 29852733 10.1021/acs.joc.8b00795

[25] D. Wutz , D. Gluhacevic , A. Chakrabarti , K. Schmidtkunz , D. Robaa , F. Erdmann , C. Romier , W. Sippl , M. Jung , B. König , Org. Biomol. Chem. 2017, 15, 4882–4896. 28537315 10.1039/c7ob00976c

[26] D. Lachmann , C. Studte , B. Männel , H. Hübner , P. Gmeiner , B. König , Chem. Eur. J. 2017, 23, 13423–13434. 28650111 10.1002/chem.201702147

[27] L. Guandalini , C. Cellai , A. Laurenzana , S. Scapecchi , F. Paoletti , M.N. Romanelli , Bioorg. Med. Chem. Lett. 2008, 18, 5071–5074. 18723349 10.1016/j.bmcl.2008.07.119

[28] H. Stobbe , Ber. Dtsch. Chem. Ges. 1907, 40, 3372–3382.

[29] H. Stobbe , Ber. Dtsch. Chem. Ges. 1905, 38, 3673–3682.

[30] H. Stobbe , Justus Liebigs Ann. Chem. 1911, 380, 1–2.

[31] K. Gewald , E. Schinke , H. Böttcher , Chem. Ber. 1966, 99, 94–100.

[32] J. Backes , E. Brunner , W. Eberbach , A. C. J. Gossauer , Houben–Weyl Methods of Organic Chemistry Vol. E 6a, Supplement: Hetarenes I (Five-Membered Rings with One Heteroatom in the Ring System), Thieme, Heidelberg, 2014.

[33] C. Valant , L. Aurelio , S. M. Devine , T. D. Ashton , J. M. White , P. M. Sexton , A. Christopoulos , P. J. Scammells , J. Med. Chem. 2012, 55, 2367–2375. 22315963 10.1021/jm201600e

[34] A. C. May , W. Fleischer , O. Kletke , H. L. Haas , O. A. Sergeeva , Br. J. Pharmacol. 2013, 170, 222–232. 23799902 10.1111/bph.12280 PMC3764863

[35] R. J. Walters , S. H. Hadley , K. D. W. Morris , J. Amin , Nat. Neurosci. 2000, 3, 1274–1281. 11100148 10.1038/81800

[36] S. P. Alexander , J. A. Peters , E. Kelly , N. Marrion , H. E. Benson , E. Faccenda , A. J. Pawson , J. L. Sharman , C. Southan , J. A. Davies , Br. J. Pharmacol. 2015, 172, 5870–5903. 26650440 10.1111/bph.13350 PMC4718212

[37] G. A. R. Johnston , M. Chebib , J. R. Hanrahan , K. N. Mewett , Curr. Drug Targets 2003, 2, 260–268. 10.2174/1568007033482805 12871036

[38] E. Engin , R. S. Benham , U. Rudolph , Trends Pharmacol. Sci. 2018, 39, 710–732. 29903580 10.1016/j.tips.2018.04.003 PMC6056379

[39] K. R. Tan , U. Rudolph , C. Lüscher , Trends Neurosci. 2011, 34, 188–197. 21353710 10.1016/j.tins.2011.01.004 PMC4020178

[40] W. Sieghart , Adv. Pharmacol. 2015, 72, 53–96. 25600367 10.1016/bs.apha.2014.10.002

