# Supplementary material for: Optical Control of GABAA Receptors with a Fulgimide‐Based Potentiator
Source: Chemistry. 2020 Sep 11;26(56):12722–7. doi: 10.1002/chem.202000710 (PMC7589408; doi:10.1002/chem.202000710)
Supplement: Supplementary file 1 — Supplementary [file CHEM-26-12722-s001.pdf]

Chemistry–A European Journal

Supporting Information

Optical Control of $GABA_A$ Receptors with a Fulgimide-Based Potentiator**

Karin Rustler+,[a] Galyna Maleeva+,[b, c] Alexandre M. J. Gomila+,[c] Pau Gorostiza,*[c, d, e] Piotr Bregestovski,*[b, f, g] and Burkhard König*[a]

Supplementary Information and Methodology

Photochemical properties 1
UV-Vis absorption spectra and cycle performance (Compound 3) 1
UV-Vis absorption spectra and cycle performance (Compound 4) 2
^{1}H- and ^{13}C-NMR spectra 4
Compound 3a 4
Compound 4a 5
Compound 7 6
Compound 9 7
Analytical HPLC traces for purity determination 8
Compound 3a (0.5 mM solution in DMSO, injection volume 3 µL) 8
Compound 4a (0.5 mM solution in DMSO, injection volume 3 µL) 9
Analytical HPLC traces for PSS determination 10
Compound 3 (0.5 mM solution in DMSO, injection volume 3 µL) 10
Compound 4 (0.5 mM solution in DMSO, injection volume 10 µL) 11
X-Ray structures 12
Compound 7 12
Additional *in vitro* Patch-Clamp data of compound 3 14
Experimental Part 15
General Procedures and Materials 15
Synthetic Procedures and Characterization 15
In Vitro Studies 16
Behavioral Studies 17
References 19

Photochemical properties

The photochromic properties of compounds **3** and **4** measured 50 µM in DMSO are summarized in table 1. The photostationary states were determined via analytical HPLC measurement of an illuminated sample and detected at the wavelength of the isosbestic point.

Table 1. Photochemical properties of fulgimide-based benzodiazepine derivatives **3** and **4** measured 50 µM in DMSO at 25 °C. Cpd = Compound. PSS = Photostationary state.

Entry	Cpd	λ_{max} open [nm]	λ_{max} closed [nm]	Isosbestic point [nm]	PSS
1	**3**	-	521	375	95% closed (UV); 99% opened (green)
2	**4**	335,347	518	377	93% closed (UV); 99% opened (green)

UV-Vis absorption spectra and cycle performance (Compound 3)

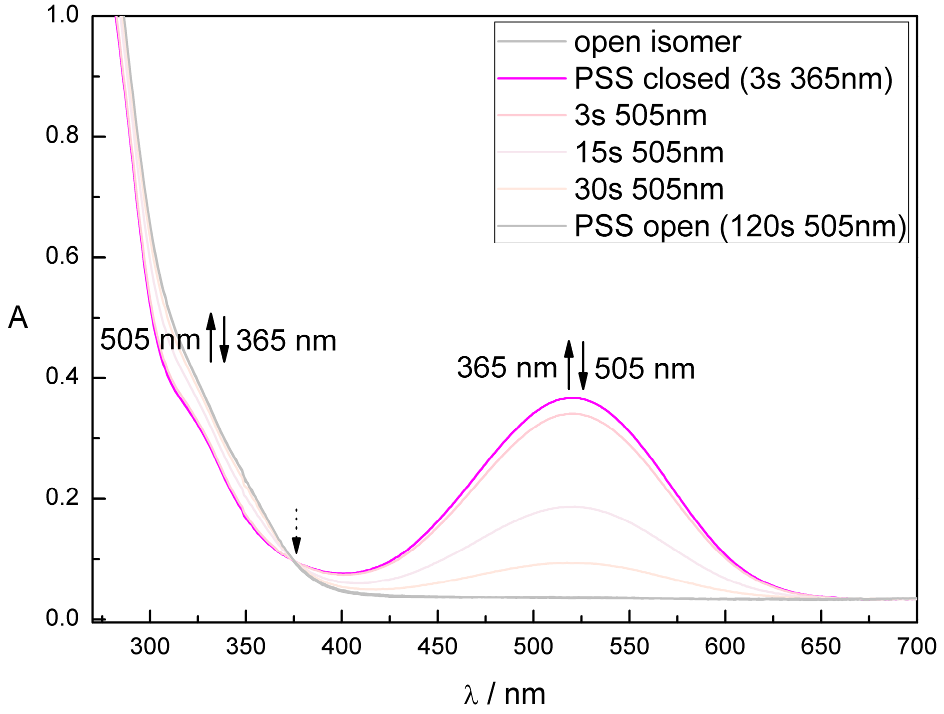

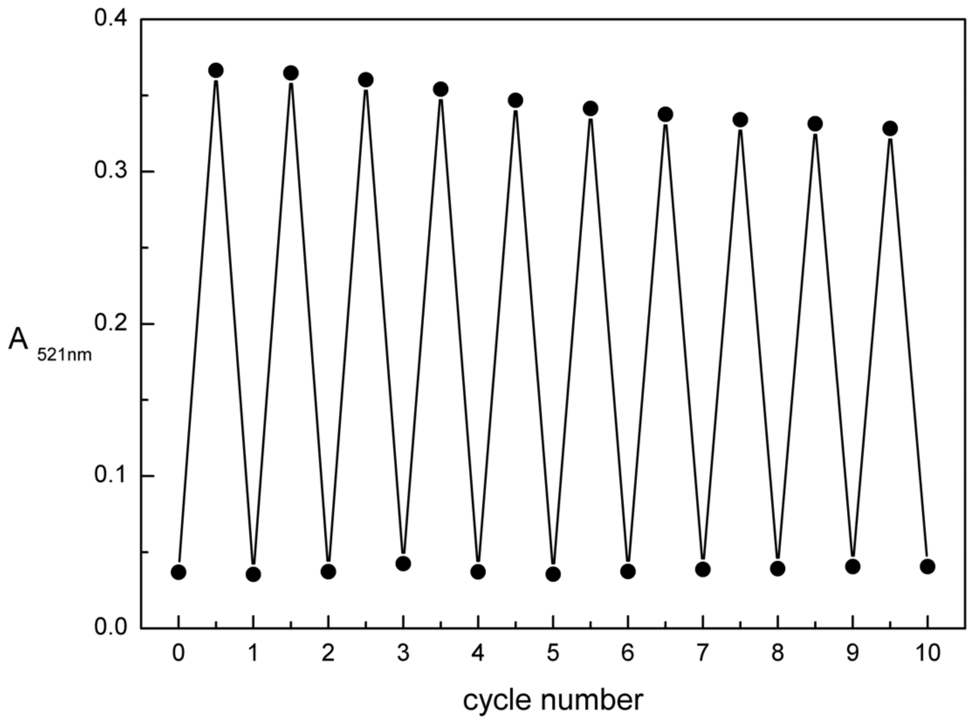

Figure S1. Upper panel. UV-Vis absorption spectrum of fulgimide **3**. Transformation of the colorless ring-open isomer (**3a**) to its purple ring-closed isomer (**3b**) upon irradiation with 365 nm. Reopening upon irradiation with 505 nm. Lower panel. Repetitive cycle performance of **3** upon alternate irradiation with 365 nm and 505 nm.

UV-Vis absorption spectra and cycle performance (Compound 4)

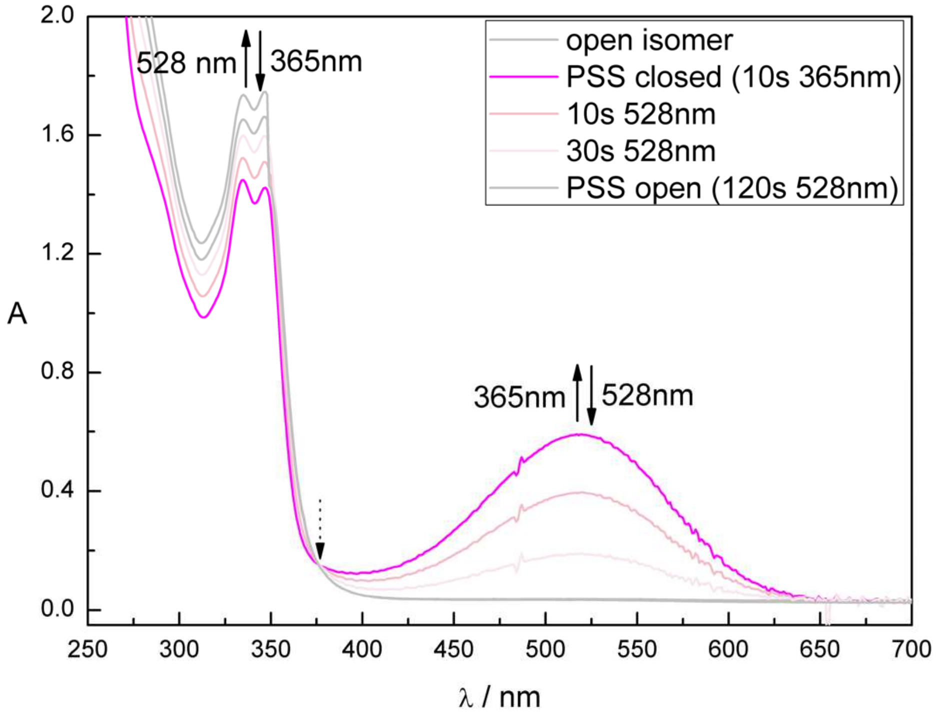

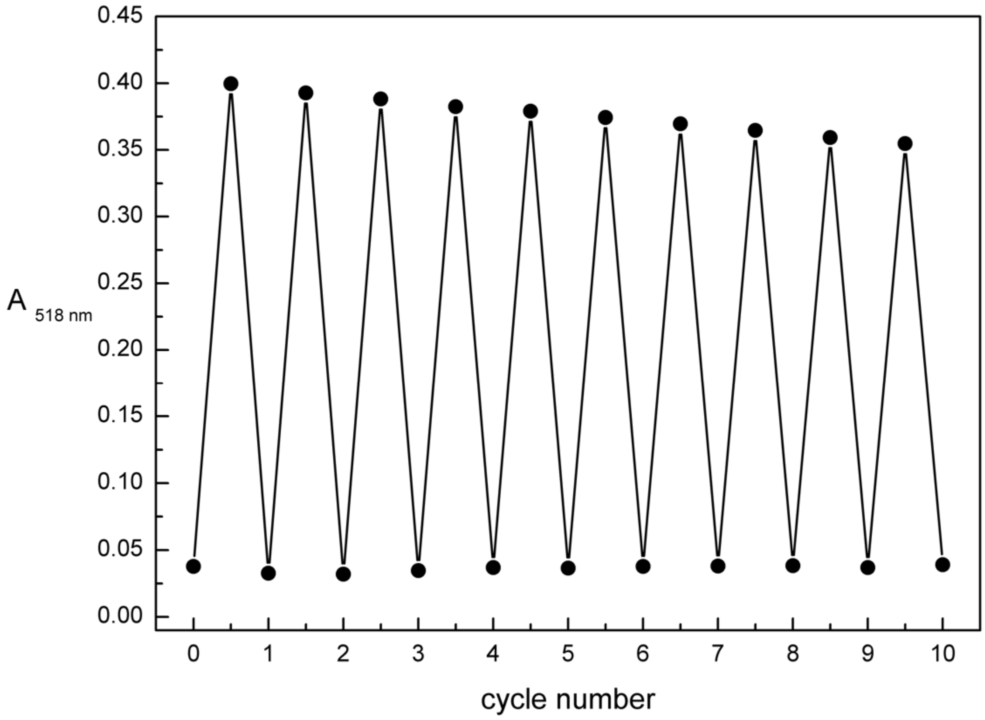

Figure S2. Upper panel. UV-Vis absorption spectrum of iso-fulgimide **4**. Transformation of the colorless ring-open isomer (**4a**) to its purple ring-closed isomer (**4b**) upon irradiation with 365 nm. Reopening upon irradiation with 528 nm. Lower panel. Repetitive cycle performance of **4** upon alternate irradiation with 365 nm and 528 nm.

^{1}H- and ^{13}C-NMR spectra

Compound 3a

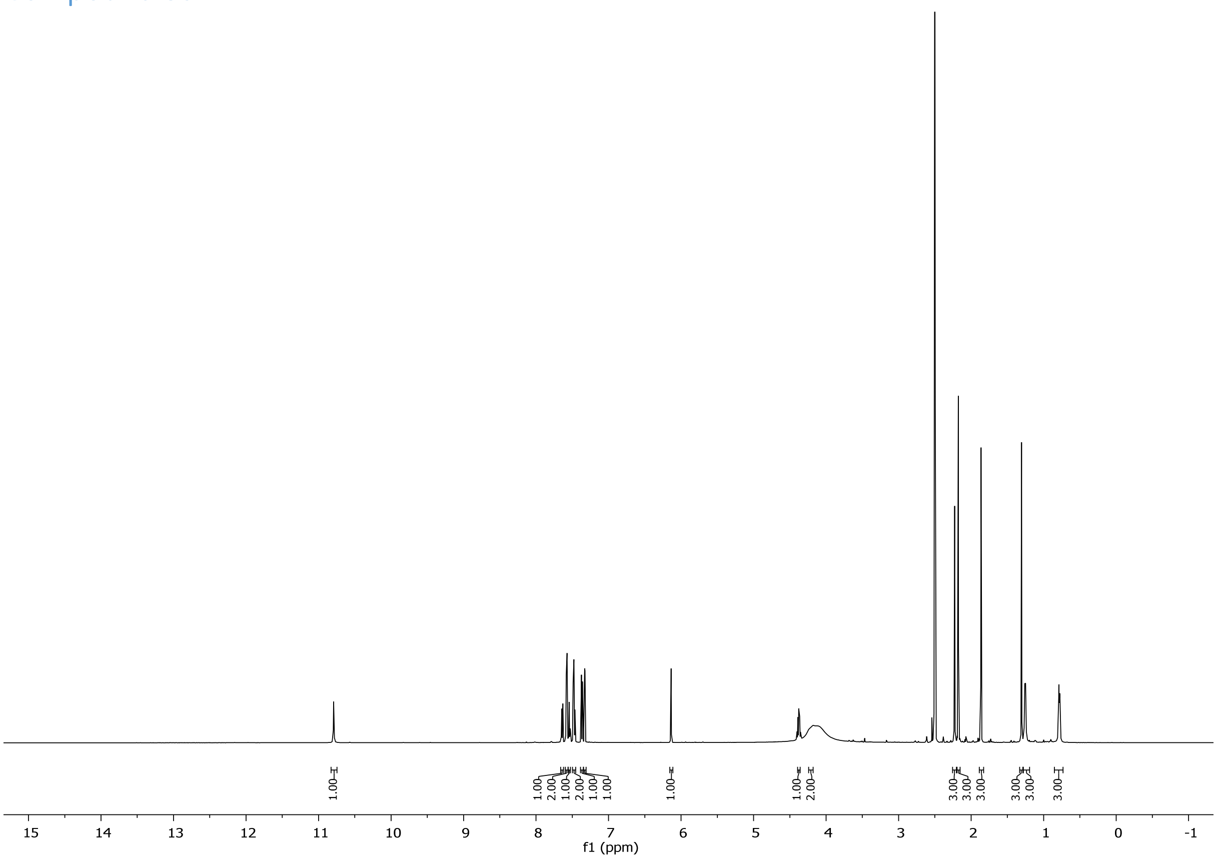

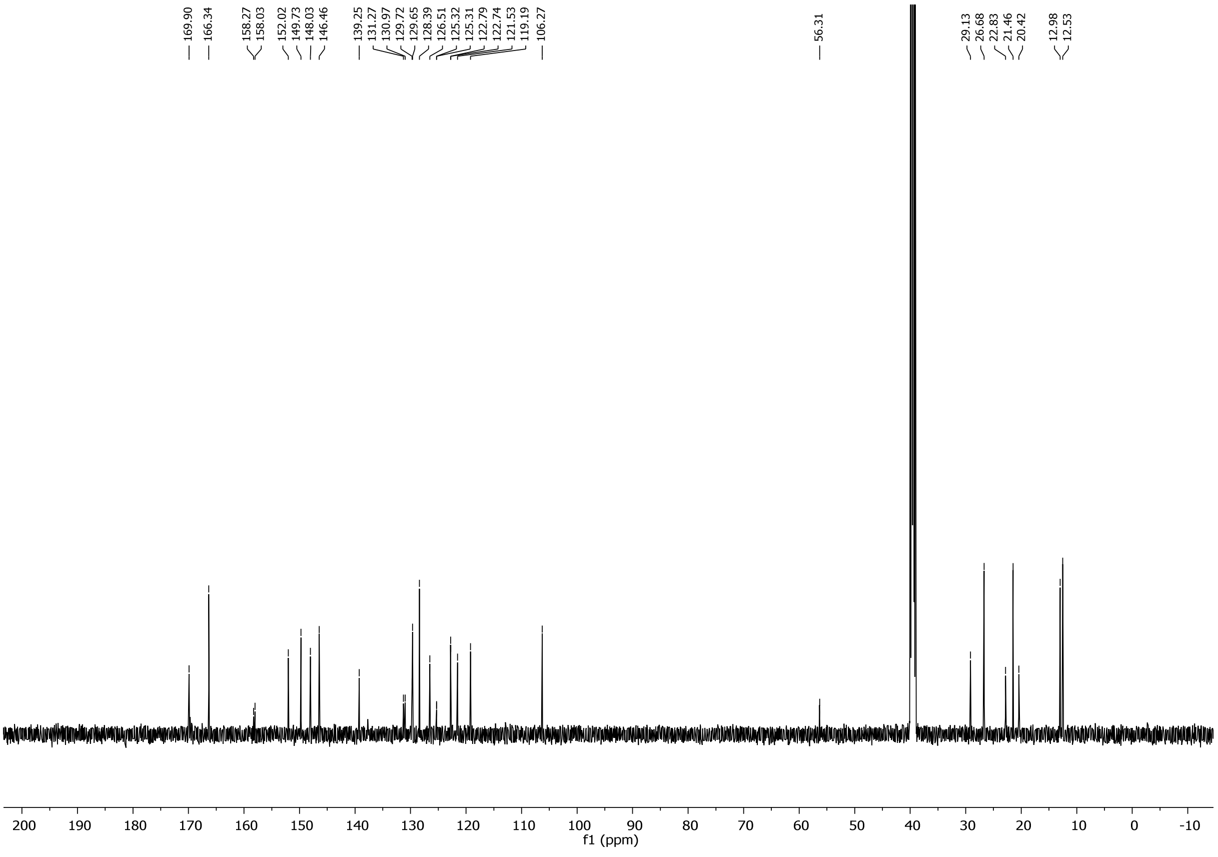

Compound 4a

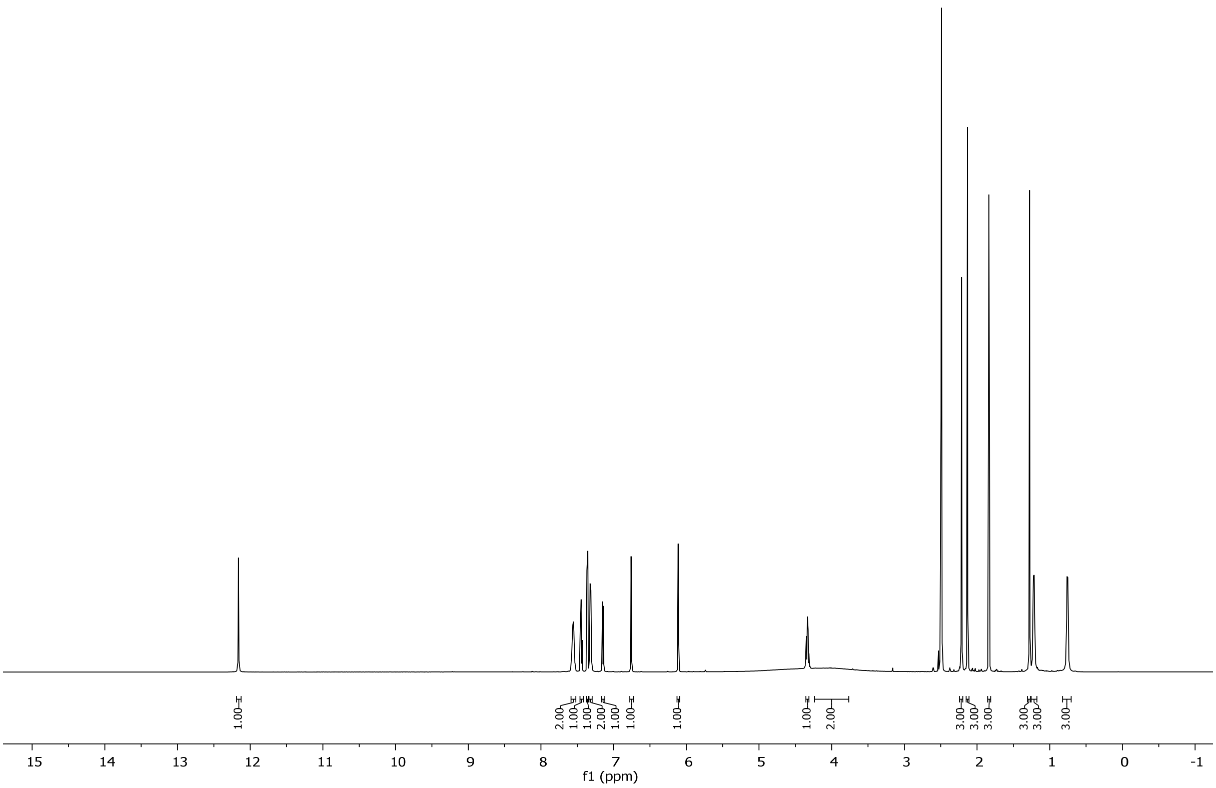
1.00
2.00
1.00
1.00
2.00
1.00
1.00
1.00
1.00
2.00
3.00
3.00
3.00
3.00
3.00
3.00
15 14 13 12 11 10 9 8 7 6 5 4 3 2 1 0 -1
f1 (ppm)

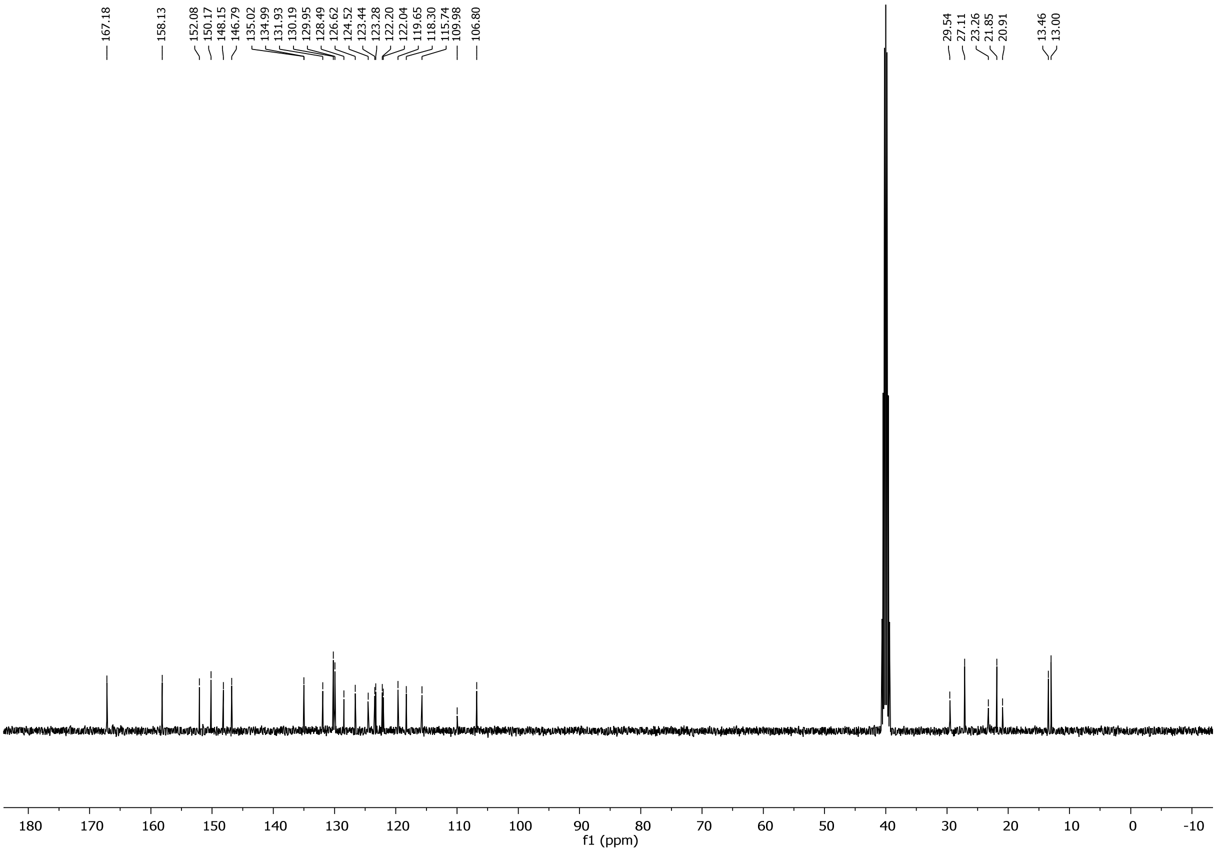
167.18
158.13
152.08
150.17
148.15
146.79
135.02
134.99
131.93
130.19
129.95
128.49
126.62
124.52
123.44
123.28
122.20
122.04
119.65
118.30
115.74
109.98
106.80
29.54
27.11
23.26
21.85
20.91
13.46
13.00
180 170 160 150 140 130 120 110 100 90 80 70 60 50 40 30 20 10 0 -10
f1 (ppm)

Compound 7

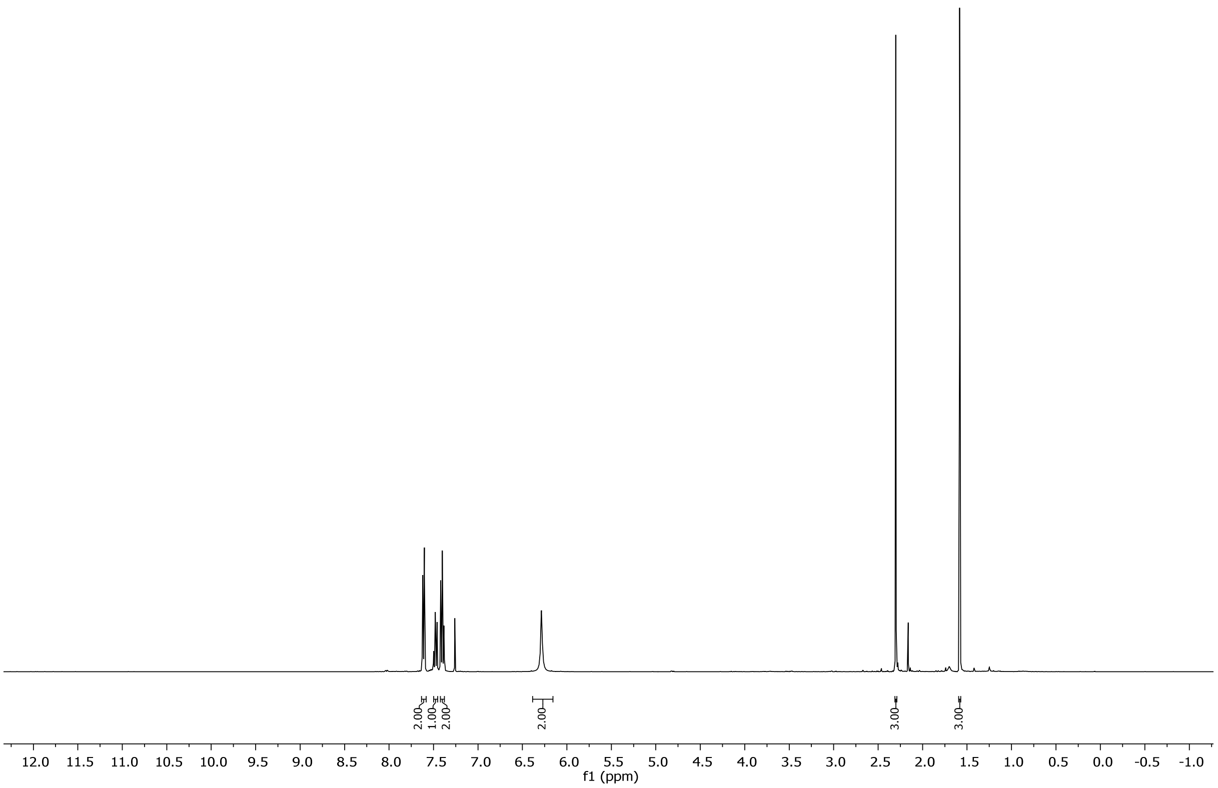
2.00
1.00
2.00
2.00
3.00
3.00
12.0 11.5 11.0 10.5 10.0 9.5 9.0 8.5 8.0 7.5 7.0 6.5 6.0 5.5 5.0 4.5 4.0 3.5 3.0 2.5 2.0 1.5 1.0 0.5 0.0 -0.5 -1.0
f1 (ppm)

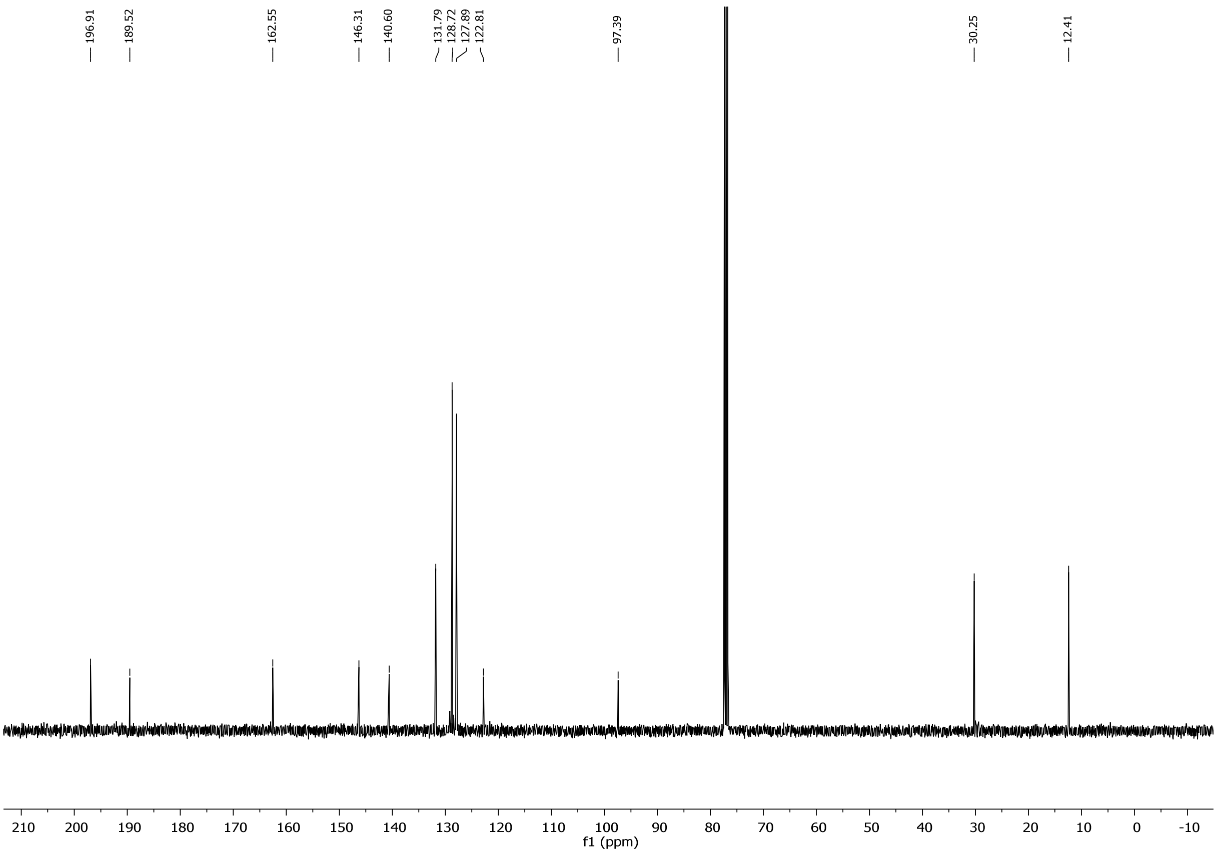
196.91
189.52
162.55
146.31
140.60
131.79
128.72
127.89
122.81
97.39
30.25
12.41
210 200 190 180 170 160 150 140 130 120 110 100 90 80 70 60 50 40 30 20 10 0 -10
f1 (ppm)

Compound 9

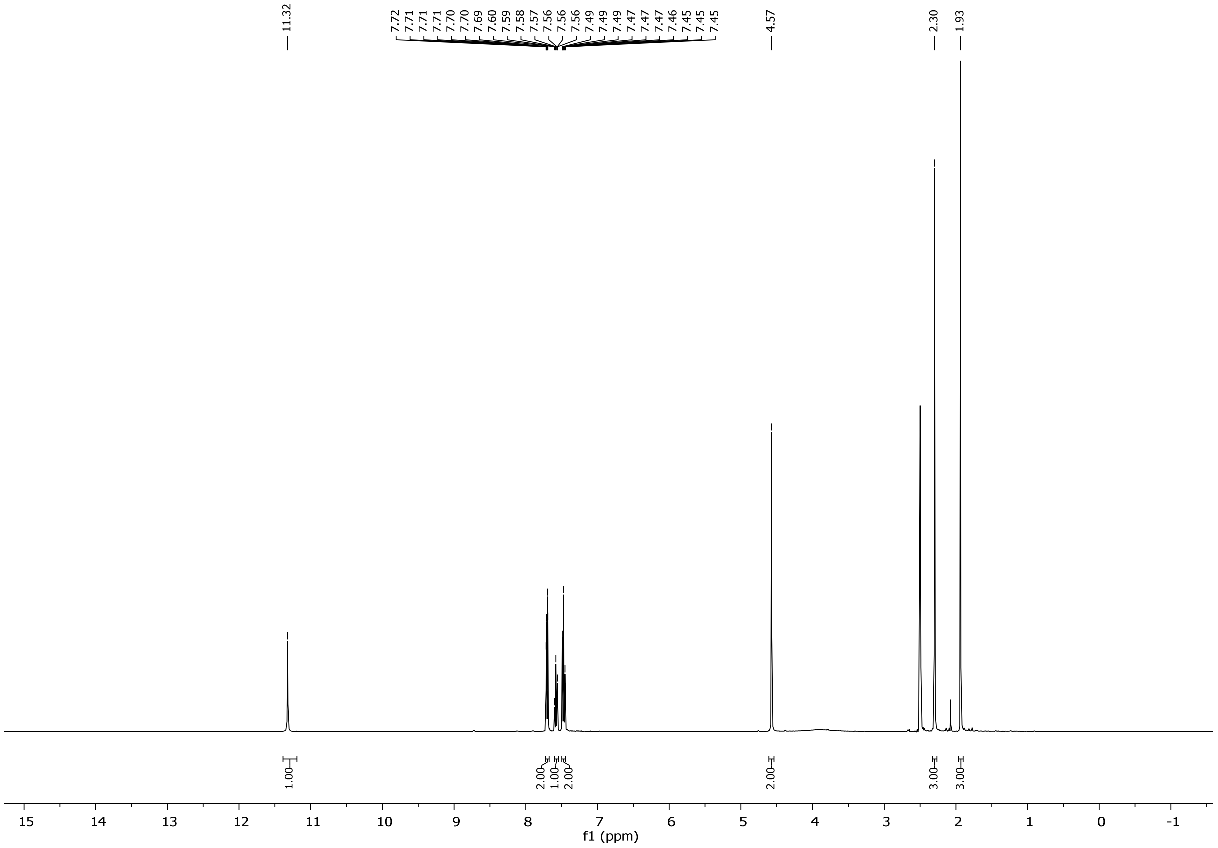
11.32
7.72
7.71
7.71
7.71
7.70
7.70
7.69
7.60
7.59
7.58
7.57
7.56
7.56
7.56
7.49
7.49
7.49
7.47
7.47
7.47
7.46
7.45
7.45
7.45
4.57
2.30
1.93
1.00
2.00
1.00
2.00
2.00
3.00
3.00
f1 (ppm)

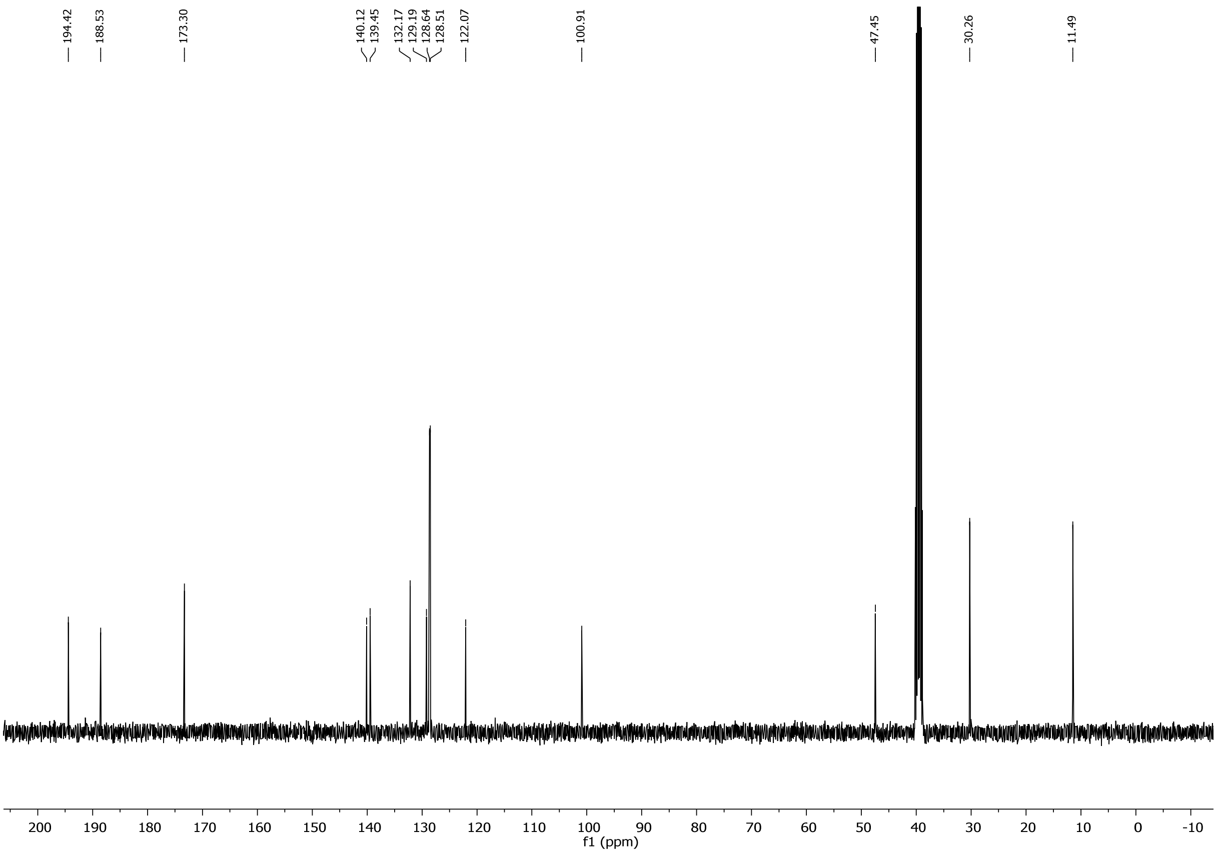
194.42
188.53
173.30
140.12
139.45
132.17
129.19
128.64
128.51
122.07
100.91
47.45
30.26
11.49
f1 (ppm)

Analytical HPLC traces for purity determination

Compound 3a (0.5 mM solution in DMSO, injection volume 3 µL)

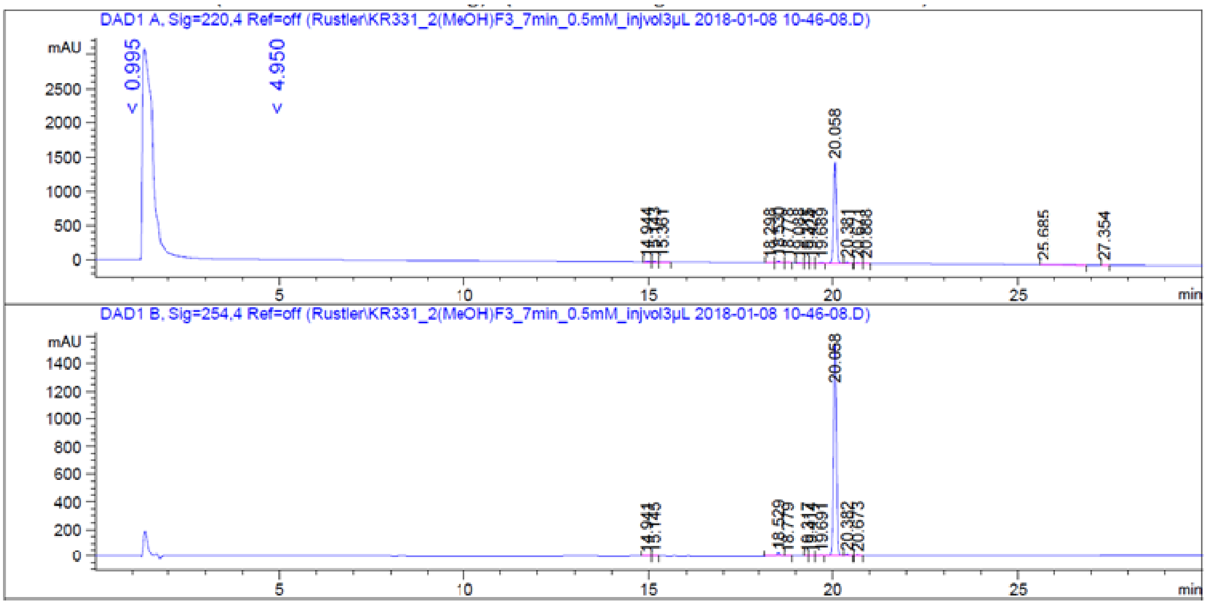

Detection at 220 nm: 95% purity

Signal 1: DAD1 A, Sig=220,4 Ref=off

Peak #	RetTime [min]	Type	Width [min]	Area [mAU*s]	Height [mAU]	Area %
1	14.944	BV	0.1043	20.30099	2.74506	0.2528
2	15.143	VV	0.0929	24.00655	3.95144	0.2989
3	15.361	VB	0.0877	10.76981	1.85496	0.1341
4	18.298	BV	0.0894	16.78782	2.90794	0.2091
5	18.530	VV	0.0819	114.85024	21.65755	1.4302
6	18.778	VB	0.0815	40.79560	7.73744	0.5080
7	19.088	BB	0.1041	9.67722	1.52325	0.1205
8	19.315	BV	0.0854	11.43824	2.03939	0.1424
9	19.424	VB	0.0793	7.09037	1.39420	0.0883
10	19.689	BV T	0.1034	24.48545	3.59901	0.3049
11	20.058	VV R	0.0810	7623.84668	1457.50513	94.9382
12	20.381	VB T	0.0765	59.39925	12.26160	0.7397
13	20.671	BB	0.0790	38.39948	7.58809	0.4782
14	20.888	BB	0.0795	6.37989	1.25011	0.0794
15	25.685	BB	0.1774	16.28838	1.20757	0.2028
16	27.354	BBA	0.0852	5.81392	1.07282	0.0724
Totals :				8030.32991	1530.29553	

Detection at 254 nm: 97% purity

Signal 2: DAD1 B, Sig=254,4 Ref=off

Peak #	RetTime [min]	Type	Width [min]	Area [mAU*s]	Height [mAU]	Area %
1	14.941	BV	0.0950	7.74013	1.17203	0.0926
2	15.145	VB	0.0855	6.42651	1.18136	0.0769
3	18.529	VV R	0.0774	127.75799	25.09272	1.5282
4	18.779	VB T	0.0794	10.23114	2.07885	0.1224
5	19.317	BV	0.0767	5.97865	1.18849	0.0715
6	19.414	VB	0.0866	7.49186	1.31197	0.0896
7	19.691	BV T	0.0981	22.28177	3.41614	0.2665
8	20.058	VV R	0.0811	8087.13916	1545.29724	96.7343
9	20.382	VB T	0.0760	51.49631	10.73422	0.6160
10	20.673	BB	0.0771	33.61602	6.63975	0.4021
Totals :				8360.15953	1598.11276	

Compound 4a (0.5 mM solution in DMSO, injection volume 3 µL)

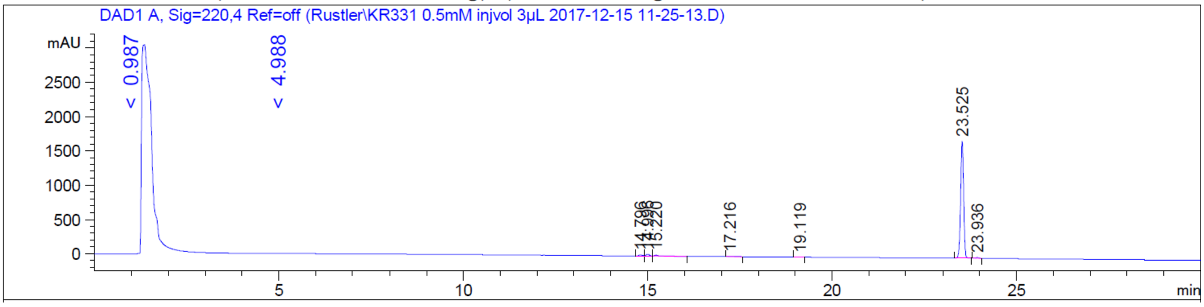

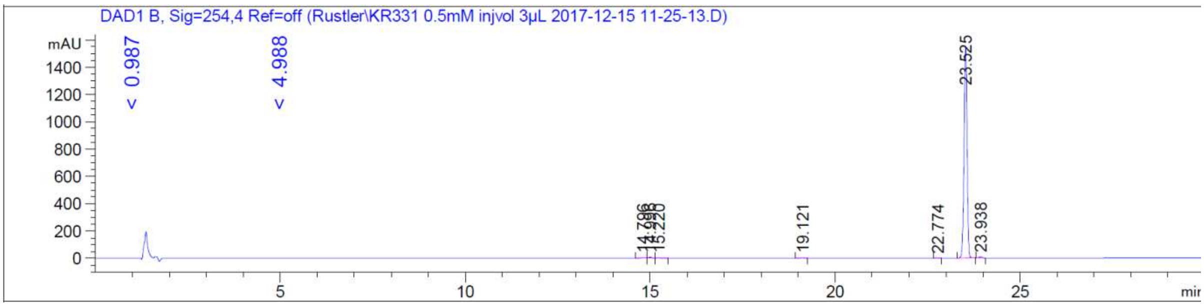

Detection at 220 nm: 95% purity

Signal 1: DAD1 A, Sig=220,4 Ref=off

Peak #	RetTime [min]	Type	Width [min]	Area [mAU*s]	Height [mAU]	Area %
1	14.796	BV	0.1162	118.11269	14.32794	1.1939
2	14.996	VV	0.0994	173.31990	26.13495	1.7519
3	15.220	VB	0.1076	95.90490	13.07767	0.9694
4	17.216	BB	0.1015	11.20386	1.60394	0.1132
5	19.119	BB	0.0875	21.09451	3.64496	0.2132
6	23.525	BB	0.0864	9418.38379	1704.98877	95.1999
7	23.936	BB	0.0900	55.24893	9.48264	0.5584
Totals :				9893.26858	1773.26088	

Detection at 254 nm: 98% purity

Signal 2: DAD1 B, Sig=254,4 Ref=off

Peak #	RetTime [min]	Type	Width [min]	Area [mAU*s]	Height [mAU]	Area %
1	14.796	BV	0.1202	44.76250	5.21297	0.5071
2	14.996	VV	0.0990	55.74607	8.44625	0.6315
3	15.220	VB	0.0949	26.13252	4.18356	0.2960
4	19.121	BB	0.0875	23.32179	4.03348	0.2642
5	22.774	BB	0.0851	8.56763	1.58528	0.0971
6	23.525	BB	0.0865	8611.23633	1557.81189	97.5530
7	23.938	BB	0.0879	57.47643	10.17249	0.6511
Totals :				8827.24325	1591.44591	

Analytical HPLC traces for PSS determination

Compound 3 (0.5 mM solution in DMSO, injection volume 3 µL)

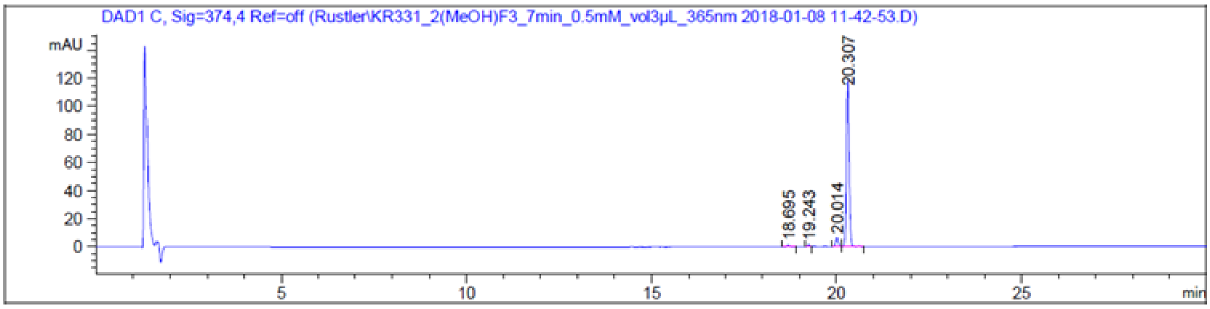

Irradiation with λ = 365 nm for conversion from open to closed state.

Detection at 374 nm: t_R open = 20.0 min (5%), t_R closed = 20.3 min (93%)

Signal 3: DAD1 C, Sig=374,4 Ref=off

Peak #	RetTime [min]	Type	Width [min]	Area [mAU*s]	Height [mAU]	Area %
1	18.695	BB	0.0741	5.67112	1.17847	0.8531
2	19.243	BB	0.0725	6.11693	1.30850	0.9202
3	20.014	BV	0.0802	33.22660	6.44082	4.9983
4	20.307	VV R	0.0811	619.74103	118.31590	93.2284
Totals :				664.75568	127.24368	

Irradiation with λ = 505 nm for conversion from closed to open state.

Detection at 374 nm: t_R open = 20.0 min (99%)

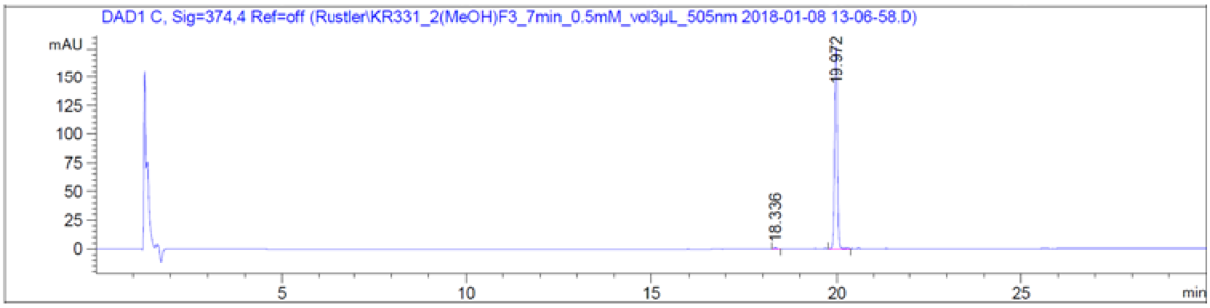

Signal 3: DAD1 C, Sig=374,4 Ref=off

Peak #	RetTime [min]	Type	Width [min]	Area [mAU*s]	Height [mAU]	Area %
1	18.336	BB	0.0684	5.76978	1.28472	0.6268
2	19.972	BV R	0.0782	914.68585	177.40359	99.3732
Totals :				920.45563	178.68832	

Compound 4 (0.5 mM solution in DMSO, injection volume 10 µL)

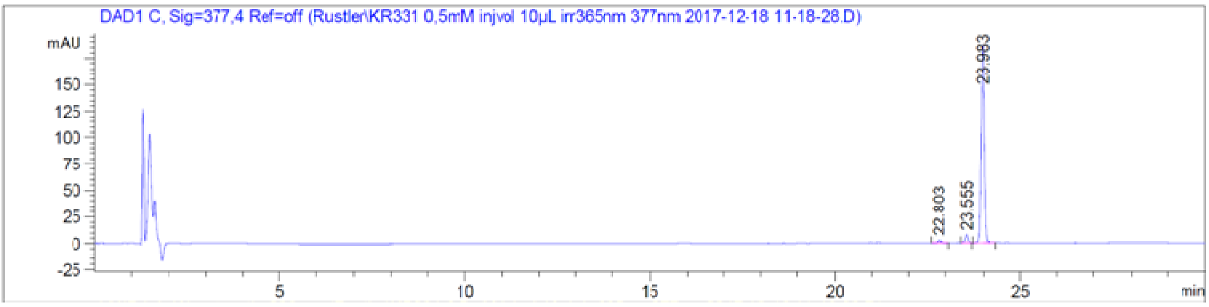

Irradiation with λ = 365 nm for conversion from open to closed state.

Detection at 377 nm: t_R open = 23.6 min (4%), t_R closed = 24.0 min (95%)

Signal 1: DAD1 C, Sig=377,4 Ref=off

Peak #	RetTime [min]	Type	Width [min]	Area [mAU*s]	Height [mAU]	Area %
1	22.803	BB	0.1073	16.79935	2.24548	1.5172
2	23.555	BB	0.0860	40.16666	7.32464	3.6276
3	23.983	BB	0.0874	1050.27759	187.22672	94.8552
Totals :				1107.24361	196.79684	

Irradiation with λ = 505 nm for conversion from closed to open state.

Detection at 374 nm: t_R open = 23.6 min (100%)

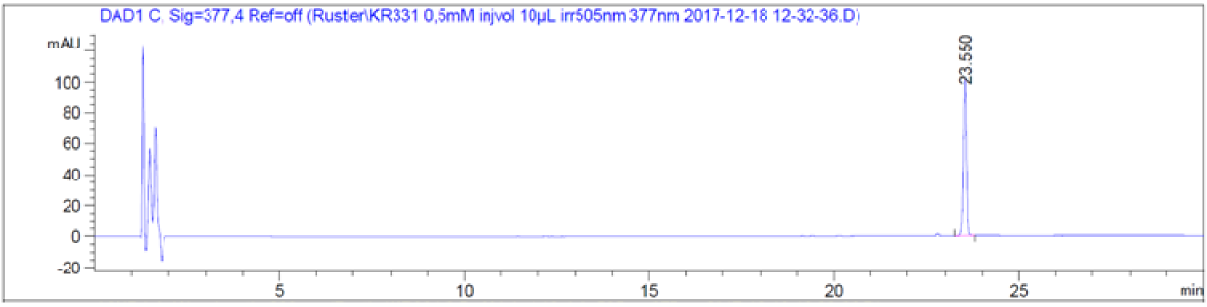

Signal 1: DAD1 C, Sig=377,4 Ref=off

Peak #	RetTime [min]	Type	Width [min]	Area [mAU*s]	Height [mAU]	Area %
1	23.550	BB	0.0853	571.35895	102.11843	100.0000
Totals :				571.35895	102.11843	

X-Ray structures

Compound 7

Experimental. Single metallic yellow prism-shaped crystals of **7** were obtained by recrystallisation from acetone. A suitable crystal 0.23×0.18×0.14 mm^3 was selected and mounted on a MITIGEN holder oil on an GV1000, TitanS2 diffractometer. The crystal was kept at a steady T = 123.0 (3) K during data collection. The structure was solved with the **ShelXT** (Sheldrick, 2015)[S1] structure solution program using the Intrinsic Phasing solution method and by using **Olex2** (Dolomanov et al., 2009)[S2] as the graphical interface. The model was refined with version 2016/6 of **ShelXL** (Sheldrick, 2015)[S3] using Least Squares minimisation.

Crystal Data. $C_{14}H_{15}NO_4$, M_r = 261.27, monoclinic, $P2_1/c$ (No. 14), a = 16.7096(5) Å, b = 13.2785(2) Å, c = 7.0935(2) Å, β = 126.945(4)°, $\alpha = \gamma$ = 90°, V = 1257.88(8) Å^3, T = 123.0(3) K, Z = 4, Z' = 1, μ(Cu K) = 0.613, 14196 reflections measured, 2534 unique (R_{int} = 0.0216) which were used in all calculations. The final wR_2 was 0.0879 (all data) and R_1 was 0.0333 (I > 2(I)).

Cambridge Structural Database CCDC: 1942472

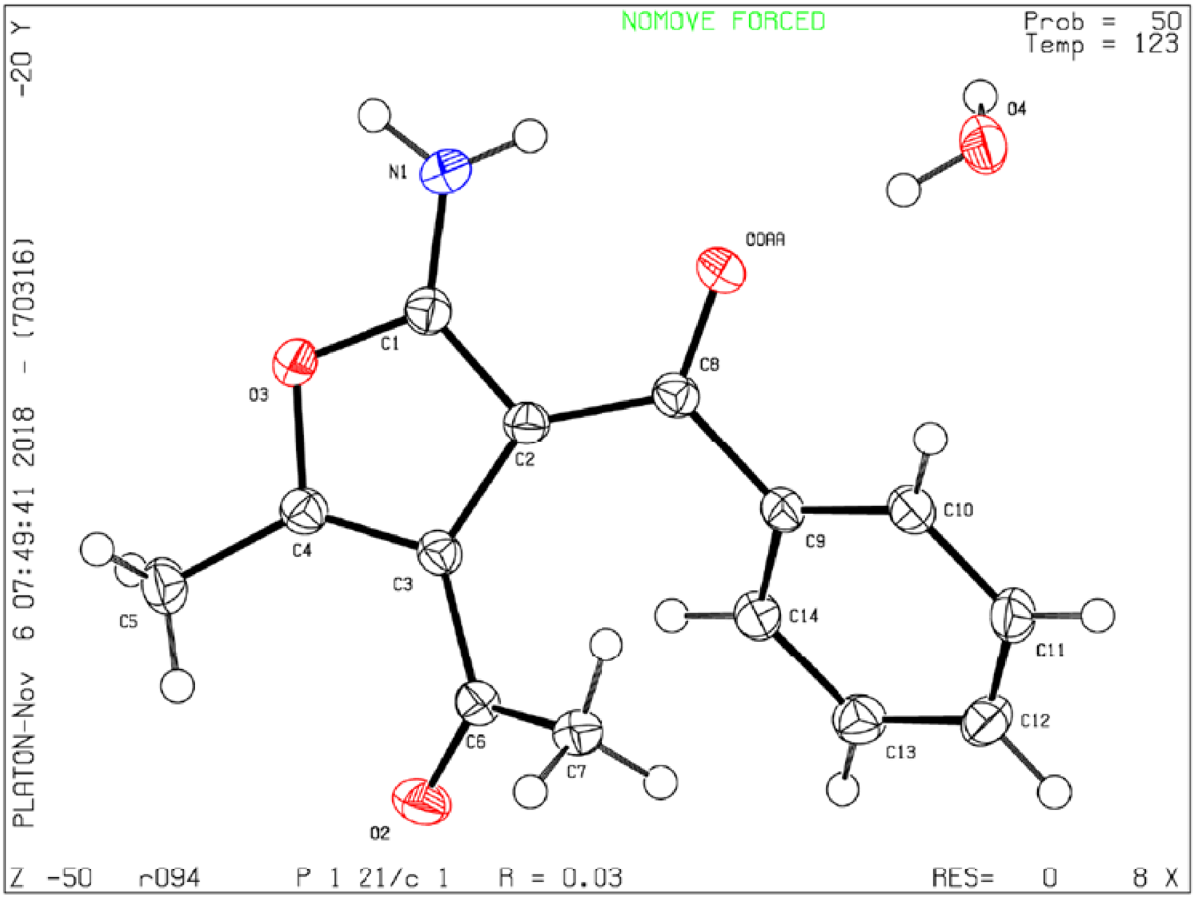

Detailed Crystal Data.

Formula	$C_{14}H_{15}NO_4$
$D_{calc.}$/ g cm^{-3}	1.380
μ/mm^{-1}	0.613
Formula Weight	261.27
Colour	metallic yellow
Shape	prism
Size/mm^3	0.23×0.18×0.14
T/K	123.0(3)
Crystal System	monoclinic
Space Group	$P2_1/c$
a/Å	16.7096(5)
b/Å	13.2785(2)
c/Å	7.0935(2)
α/°	90
β/°	126.945(4)
γ/°	90
V/Å^3	1257.88(8)
Z	4
Z'	1
Wavelength/Å	1.39222
Radiation type	Cu K
Θ_{min}/°	4.240
Θ_{max}/°	60.099
Measured Refl.	14196
Independent Refl.	2534
Reflections with I > 2(I)	2348
R_{int}	0.0216
Parameters	178
Restraints	0
Largest Peak	0.305
Deepest Hole	-0.217
GooF	1.037
wR_2 (all data)	0.0879
wR_2	0.0862
R_1 (all data)	0.0354
R_1	0.0333

Additional *in vitro* Patch-Clamp data of compound 3

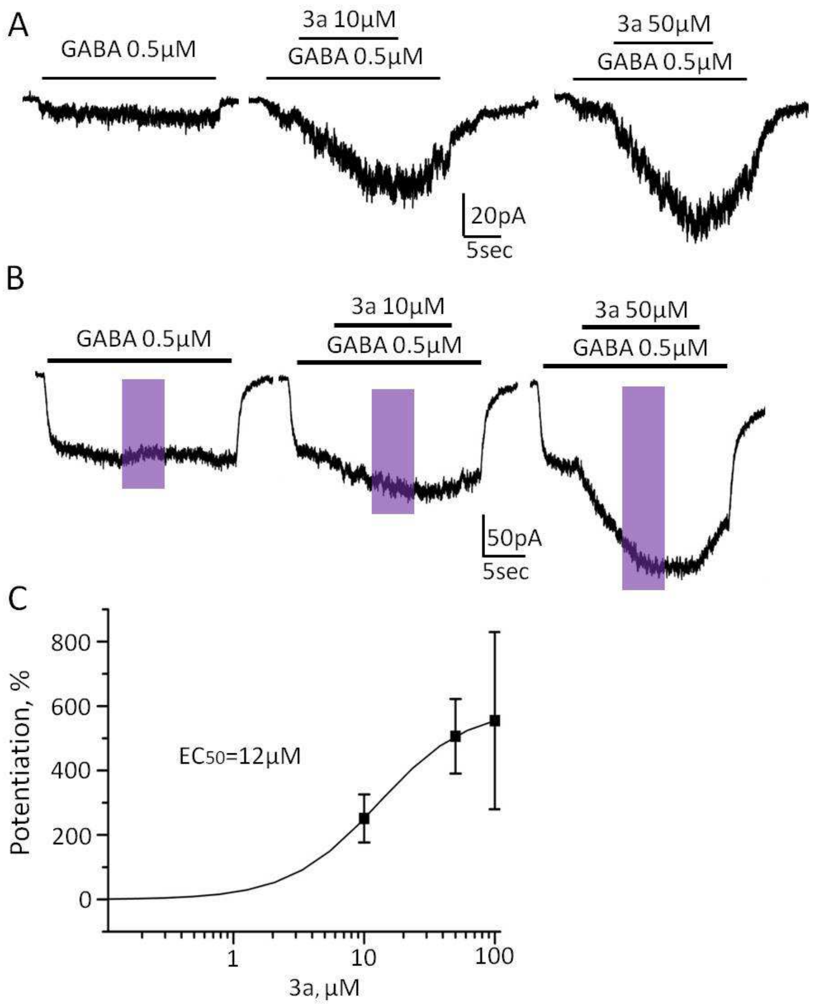

Figure S3. The effect of **3** on $GABA_A$-mediated currents upon application in its open state (**3a**) and illumination with UV light for light-triggered ring-closing (highlighted in purple) (**3b**). **A.** Representative traces of currents induced by application of GABA 0.5 μM (left), by mixture of GABA 0.5 μM with **3a** 10 μM (center) and by mixture of GABA 0.5 μM with **3a** 50 μM (right). **B.** Representative traces demonstrating the absence of the effect of UV light illumination (triggering ring closing) on the amplitude of currents induced by application of GABA 0.5 μM (left), by mixture of GABA with **3a** 10 μM (center) and by mixture of GABA 0.5 μM with **3a** 50 μM (right). **C.** Cumulative dose-response curve for the compound **3a** (n=4-11).

Experimental Part

General Procedures and Materials

Commercial reagents and starting materials were purchased from the commercial suppliers abcr, Acros Organics, Alfa-Aesar, Fisher Scientific, Merck, Sigma Aldrich, TCI, or VWR and used without any further purification. Solvents were used in p.a. quality and dried according to common procedures, if necessary. Dry nitrogen was used as an inert gas atmosphere. Flash column chromatography was performed using Sigma Aldrich MN silica gel 60 M (40-63 µm, 230-400 mesh) for normal phase chromatography. Reaction monitoring *via* thin layer chromatography was performed on alumina plates coated with silica gel (Merck silica gel 60 F_{254}, layer thickness 0.2 mm). Melting points were determined using a Stanford Research System OptiMelt MPA 100 and are uncorrected. NMR spectra were measured on a Bruker Avance 300 (^{1}H 300.13 MHz, ^{13}C 75.48 MHz), Bruker Avance III HD 400 (^{1}H 400.13 MHz, ^{13}C 100.61 MHz), Bruker Avance III HD 600 (^{1}H 600.25 MHz, ^{13}C 150.95 MHz) and Bruker Avance III 600 (^{1}H 600.25 MHz, ^{13}C 150.95 MHz). The spectra are referenced against the NMR-solvent (DMSO-*d*$_6$: δ_H = 2.50 ppm, δ_C = 39.52 ppm; $CDCl_3$-*d*: δ_H = 7.26 ppm, δ_C = 77.16 ppm) and chemical shifts δ are reported in ppm. Resonance multiplicity is abbreviated as: s (singlet), d (doublet), t (triplet) and m (multiplet). Carbon NMR signals are assigned using DEPT 135 and ^{1}H-^{13}C HSQC spectra with (+) for primary/tertiary, (-) for secondary, and (q) for quaternary carbons. Mass spectra were recorded on a Finnigan MAT-SSQ 710 A, ThermoQuest Finnigan TSQ 7000, Agilent Q-TOF 6540 UHD, or a JeolAccuTOF GCX instrument. UV-Vis absorption spectroscopy was performed in 10 mm quartz cuvettes using an Agilent Cary 100 or Agilent Varian Cary 50 spectrometer. Analytical HPLC measurements were performed using an Agilent 1220 Infinity LC (column: Phenomenex Luna 3 µm C_{18}(2) 100 Å, 150 x 2.00 mm; flow 0.3 mL min^{-1} at 30 °C; solvent A: MilliQ water with 0.05 wt% TFA; solvent B: MeCN). The ratios at the PSSs were determined *via* analytical HPLC at 20 °C at the isosbestic points. An Agilent 1260 system (column: Phenomenex Luna 10 µm C_{18}(2) 100 Å, 250 x 21.2 mm; flow: 22 mL min^{-1}; solvent A: MilliQ water with 0.05 wt% TFA; solvent B: MeCN) was used for preparative HPLC purification. Light sources for irradiation: λ = 365 nm (Herolab hand-held lamp UV-6 L, 6W; Seoul Viosys CUN6GB1A, 1000 mA, 1.4 W), λ = 505 nm (Osram LVCK7P-JYKZ, 350 mA, 112 lm), λ = 528 nm (Osram LTCP7P-KXKZ, 350 mA, 71 lm). The power of the light source is given based on the specifications supplied by the company when the LEDs were purchased. All tested final compounds possess a purity ≥ 95% determined by analytical HPLC measurements with detection at 220 nm and 254 nm, respectively. Compounds **1**[S4] and **2**[S5] were synthesized following literature reported procedures.

Synthetic Procedures and Characterization

(Iso-)fulgimide synthesis. A solution of fulgide **2**[S5] (0.25 g, 0.87 mmol, 1.0 eq) and amino-nitrazepam **1**[S4] (0.24 g, 0.96 mmol, 1.1 eq) in methanol (10 mL) was heated to 60 °C and stirred for 16 h. The mixture was cooled to room temperature. Then, DCC (0.23 g, 1.1 mmol, 1.3 eq), HOBt (0.15 g, 1.1 mmol, 1.3 eq) and DIPEA (0.20 mL, 0.15 g, 1.1 mmol, 1.3 eq) were added and the mixture stirred at room temperature for three days. The reaction mixture was filtered and concentrated *in vacuo*. Purification by column chromatography (petroleum ether/ethyl acetate 1/1) and subsequent preparative HPLC (55% - 98% MeCN in 12 min) yielded fulgimide **3a** (t_R = 7.2 min, 8%) and iso-fulgimide **4a** (t_R = 11.2 min, 20%) as slightly yellow solids.

(*E*)-3-(1-(2,5-dimethylfuran-3-yl)-2-methylpropylidene)-1-(2-oxo-5-phenyl-2,3-dihydro-1*H*-benzo[*e*][1,4]diazepin-7-yl)-4-(propan-2-ylidene)pyrrolidine-2,5-di-one (3a). M.p.: 115 °C. ^{1}H-NMR (600 MHz, DMSO-*d*$_6$): δ = 10.79 (s, 1H), 7.64 (dd, *J* = 8.7, 2.3 Hz, 1H), 7.60 – 7.56 (m, 2H), 7.56 – 7.53 (m, 1H), 7.48 (m, 2H), 7.37 (d, *J* = 8.7 Hz, 1H), 7.33 (d, *J* = 2.3 Hz, 1H), 6.14 (s, 1H), 4.39 – 4.36 (m, 1H), 4.18 (s, 2H), 2.23 (s, 3H), 2.18 (s, 3H), 1.86 (s, 3H), 1.30 (s, 3H), 1.25 (d, *J* = 7.1 Hz, 3H), 0.78 (d, *J* =

6.8 Hz, 3H). ^{13}C-NMR (151 MHz, DMSO-*d$_6$*): δ = 169.9 (q), 166.3 (q), 158.3 (q), 158.0 (q), 152.0 (q), 149.7 (q), 148.0 (q), 146.5 (q), 139.3 (q), 131.3 (+), 131.0 (+), 129.7 (+), 129.7 (+), 128.4 (+), 126.5 (q), 125.3 (q), 125.3 (q), 122.8 (q), 122.7 (q), 121.5 (+), 119.2 (q), 106.3 (+), 56.3 (-), 29.1 (+), 26.7 (+), 22.8 (+), 21.5 (+), 20.4 (+), 13.0 (+), 12.5 (+). HRMS (ESI) calcd. for $C_{32}H_{31}N_3O_4Na$ $[M+Na]^+$: m/z = 544.2207, found 544.2206. MF: $C_{32}H_{31}N_3O_4$. MW: 521.62 g/mol.

7-(((2*Z*,4*E*)-4-(1-(2,5-dimethylfuran-3-yl)-2-methylpropylidene)-5-oxo-3-(pro-pan-2-ylidene)dihydrofuran-2(3*H*)-ylidene)amino)-5-phenyl-1,3-dihydro-2*H*-benzo[*e*][1,4]diazepin-2-one (4a).M.p.: 146 °C. ^{1}H-NMR (600 MHz, DMSO-*d$_6$*): δ = 12.16 (s, 1H), 7.59 – 7.52 (m, 2H), 7.45 (tt, *J* = 7.5, 1.3 Hz, 1H), 7.36 (d, *J* = 8.6 Hz, 1H), 7.32 (d, *J* = 7.5 Hz, 2H), 7.15 (dd, *J* = 8.5, 2.2 Hz, 1H), 6.76 (d, *J* = 2.1 Hz, 1H), 6.11 (s, 1H), 4.35 – 4.31 (m, 1H), 4.02 (s, 2H), 2.21 (s, 3H), 2.13 (s, 3H), 1.84 (s, 3H), 1.28 (s, 3H), 1.22 (d, *J* = 7.1 Hz, 3H), 0.75 (d, *J* = 6.8 Hz, 3H). ^{13}C-NMR (101 MHz, DMSO-*d$_6$*): δ = 167.2 (q), 158.1 (q), 152.1 (q), 150.2 (q), 148.2 (q), 146.8 (q), 135.0 (q), 135.0 (q), 131.9 (q), 130.2 (+), 123.0 (+), 128.5 (+), 126.6 (q), 124.5 (+), 123.4 (q), 123.3 (q), 122.2 (q), 122.0 (+), 119.7 (q), 118.3 (q), 115.7 (+), 110.0 (-), 106.8 (+), 29.5 (+), 27.1 (+), 23.3 (+), 21.9 (+), 20.9 (+), 13.5 (+), 13.0 (+). HRMS (ESI) calcd. for $C_{32}H_{31}N_3O_4Na$ $[M+Na]^+$: m/z = 544.2207, found 544.2210. MF: $C_{32}H_{31}N_3O_4$. MW: 521.62 g/mol.

1-(5-amino-4-benzoyl-2-methylfuran-3-yl)ethan-1-one (7). Morpholine (2.9 g, 33 mmol, 1.1 eq) was added to a suspension of benzoylacetonitrile (4.4 g, 30 mmol, 1.0 eq) in EtOH (30 mL). 3-Chloro-2,4-diketo-pentan (4.0 g, 30 mmol, 1.0 eq) was added dropwise. The solution was heated to reflux for three hours.[S6-S8] Evaporation of the solvent and purification by column chromatography (petroleum ether/ethyl acetate 1/1) afforded the desired product as yellow solid (1.5 g, 6.0 mmol, 20%). M.p.: 86 °C. ^{1}H-NMR (400 MHz, $CDCl_3$-*d*): δ = 7.65 – 7.58 (m, 2H), 7.52 – 7.45 (m, 1H), 7.42 – 7.35 (m, 2H), 6.29 (s, 2H), 2.30 (s, 3H), 1.58 (s, 3H). ^{13}C-NMR (101 MHz, $CDCl_3$-*d*): δ = 196.9 (q), 189.5 (q), 162.6 (q), 146.3 (q), 140.6 (q), 131.8 (+), 128.7 (+), 127.9 (+), 122.8 (q), 97.4 (q), 30.3 (+), 12.4 (+). HRMS (ESI) calcd. for $C_{14}H_{14}NO_3$ $[M+H]^+$: m/z = 244.0968, found 244.0967. MF: $C_{14}H_{13}NO_3$. MW: 243.26 g/mol.

6-Acetyl-7-methyl-5-phenyl-1,3-dihydro-2H-furo[2,3-e][1,4]diazepin-2-one (9*).*Compound **9** was synthesized *via* an adapted literature procedure[S4] starting from tetrasubstituted furan **7** (0.50 g, 2.1 mmol, 1.0 eq) and Fmoc-Glycine (0.61 g, 2.1 mmol, 1.0 eq). Purification by column chromatography (CH_2Cl_2 + 10% MeOH) and subsequent preparative HPLC (10% - 98% MeCN in 25 min, t_R = 10.1 min) afforded the desired product as slightly yellow solid (0.23 g, 0.80 mmol, 38% over two steps). M.p.: decomposition over 300 °C. ^{1}H-NMR (400 MHz, DMSO-*d$_6$*): δ = 11.32 (s, 1H), 7.72 – 7.67 (m, 2H), 7.60 – 7.54 (m, 1H), 7.50 – 7.45 (m, 2H), 4.57 (s, 2H), 2.30 (s, 3H), 1.93 (s, 3H). 13C-NMR (101 MHz, DMSO-*d$_6$*): δ = 194.4 (q), 188.5 (q), 173.3 (q), 140.1 (q), 139.5 (q), 132.2 (+), 129.2 (q), 128.6 (+), 128.5 (+), 122.1 (q), 100.9 (q), 47.5 (-), 30.3 (+), 11.5 (+). HRMS (ESI) calcd. for $C_{16}H_{15}N_2O_3$ $[M+H]^+$: m/z = 283.1077, found 283.1076. MF: $C_{16}H_{14}N_2O_3$. MW: 282.30 g/mol.

In Vitro Studies

Cell culture and transfection.$GABA_A$ receptors were heterologously expressed in cultured Chinese hamster ovary (CHO) cells obtained from the American Type Tissue Culture Collection (ATCC, Molsheim, France) that were maintained in culture conditions as previously described.[S9] Cells were simultaneously transfected with cDNAs of alpha1, beta2 and gamma2 subunits (concentrations 0.9-1.2 µg/µl). One day before transfection, cells were plated on the cover slips (12 mm in diameter) and placed inside 35 mm cell culture dishes with 2 ml of medium. Transfection was performed using the Lipofectamine 3000 protocol (Life Technology, USA). To facilitate identification of transfected cells a green fluorescent protein (GFP, 0.5 µg/µl) was added to the transfection mixture. Electrophysiological recordings were performed in the fluorescent cells 24-72 hours after transfection.

Electrophysiological recordings on CHOcells. Whole-cell recordings were performed at room temperature (20-25 °C) using an EPC-9 amplifier (HEKA Elektronik, Germany). Cells were continuously superfused with external solution containing (mM): NaCl 140, $CaCl_2$ 2, KCl 2.8, $MgCl_2$ 4, HEPES 20, glucose 10; pH 7.4; 320-330 mOsm. Intracellular solution used for filling recording pipettes contained (mM): KCl 140, $MgCl_2$ 2, MgATP 2, HEPES 10, BAPTA (tetrapotassium salt) 2; pH 7.3; 290 mOsm. Recording pipettes were pulled from borosilicate glass capillaries (Harvard Apparatus Ltd, USA) and had resistances of 5-10 MOhms. For the rapid replacement of the solutions, the fast application system was used. Three parallel rectangular tubes (100 x 100 µm) were positioned 40 - 50 µm above the recorded cell. The movement of the tubes was controlled by a computer-driven fast exchange system (SF 77A Perfusion Fast-Step, Warner, USA) allowing a 10–90% solution exchange in 3–5 ms, as measured by open electrode controls (1/10 external solution/water). Cells with low input resistance (<150 MOhms) and a rapid run-down (>30% with repetitive application) were excluded from analysis. Recordings were performed at holding potential (V_{hold}) of -30 mV. Pure agonist was applied during five seconds at the beginning and at the end of the trace; the mixture of the agonist with studied compounds was applied during 15 seconds in the middle of the trace. UV light (365 nm) was applied during five seconds in the middle of the trace. UV light emitting diode (Thorlabs) was placed at the distance of 4-5 cm from the recorded cell. The power of UV light was reaching 0.6 mW/mm^2, which was determined using an optical power meter (Thorlabs).

Behavioral Studies

Animal housing and photoswitchable behavioral assays. Tupfel-Lon *Danio rerio* embryos were raised in darkness for 6 days post fertilization (dpf) in UV filtered tap water in petri dishes (daily cleaned and refilled) at 28.5 °C. Larvae were recorded and video analyzed using the Zebrabox and Zebralab software (ViewPoint Life Sciences). For all experiments, 7 dpf larvae were left undisturbed for 40 minutes in 200 µL fresh UV filtered water and in darkness. Continuously, 100 µL were removed and replaced with a double concentrated treatment solution and data and video recording begun. For the first 20 minutes, larvae were kept in darkness measuring basal activity, named as the relaxation period (RP). After the RP, three double light illumination cycles were applied; 2 minutes 500 nm (visible light) and 2 minutes of darkness followed by 2 minutes of 365 nm (UV light) and 2 minutes of darkness. Hence, it was assured that solutions for compound **4** transit between their respective open (**4a**) and closed states (**4b**). As both opened and closed photostationary states are stable in dark, larvae were applied each solution independently. Original compound solution was received as a full opened state solution and was kept in dark before the addition to larvae wells. Closed state was achieved by illumination with 365 nm lamp original solution for 5 minutes before the addition as double concentrated solution to larvae. Data and video recording lasted for 48 minutes in order to acquire a RP and light transition measurements. Compounds toxicities were tested leaving 6 and 7 dpf larvae over 24 hours into different concentration solutions of each compound tested and screened for any physical abnormalities or mortality.

Data analysis and statistics. Zebrafish tracking was performed in real time and data acquisition integrated one-minute intervals using the Zebralab software (ViewPoint Life Science). Data statistical analysis were performed using GraphPad Prism 6 software. Selective illumination was performed with two ordered based [evenly distributed] arrays of 12 light emitting diodes (LEDs) for each wavelength placed 12cm afar of the multiwell plate. The light intensities, measured with an optical power meter (model Newport 1916-C), were 5.92 Wm^{-2} for 365 nm (UV) and 2.2 Wm^{-2} for 500 nm (Visible-Green). Distance activity was measured as the sum of swimming distances (in millimeters) during burst activities (larvae swimming velocities higher than 6 mm·s^{-1}) over one-minute integration (Figure 5A). Data was analyzed following two-way ANOVA with Tukey's multiple comparison test

(Figure 5B, bottom) or Bonferroni's (Figure 5B, top) and are presented as mean ± standard error of the mean (s.e.m.) or standard deviation (s.d.) with the number of larvae (*n*) indicated in each case.

References

[S1] G. M. Sheldrick, *Acta Cryst.*, **2015**, *A71*, 3-8.

[S2] O.V. Dolomanov, L.J. Bourhis, R.J. Gildea, J.A.K. Howard, H. Puschmann, *J. Appl. Cryst.***2009**, *42*, 339-341.

[S3] G. M. Sheldrick, *Acta Cryst.***2015**, *C27*, 3-8.

[S4] L. Guandalini, C. Cellai, A. Laurenzana, S. Scapecchi, F. Paoletti, M. N. Romanelli, *Bioorg. Med. Chem. Lett.***2008**, *18*, 5071-5074.

[S5] D. Wutz, D. Gluhacevic, A. Chakrabarti, K. Schmidtkunz, D. Robaa, E. Erdmann, C. Romier, W. Sippl, M. Jung, B. König, *Org. Biomol. Chem.* **2017**, *15*, 4882-4896.

[S6] K. Gewald, E. Schinke, H. Böttcher, *Chem. Ber.* **1966**, *99*, 94-100.

[S7] J. Backes, E. Brunner, W. Eberbach, A. Gossauer, C. Jutz, *Houben Weyl Methods of Organic Chemistry* **2014**, *E6a*, 227.

[S8] C. Valant, L. Aurelio, S. M. Devine, T. D. Ashton, J. M. White, P. M. Sexton, A. Christopuoulos, P. J. Scammells, *J. Med. Chem.* **2012**, *55*, 2367-2375.

[S9] G. Maleeva, S. Buldakova, P. Bregestovski, *Front. Mol. Neurosci.* **2015**, *8*, 64.
